# MiR-4310 induced by SP1 targets PTEN to promote glioma progression

**DOI:** 10.1186/s12935-020-01650-9

**Published:** 2020-12-17

**Authors:** Zhiyong Wu, Jie Luo, Tengyue Huang, Renhui Yi, Shengfeng Ding, Cheng Xie, An’qi Xu, Yu Zeng, Xizhao Wang, Ye Song, Xiaofeng Shi, Hao Long

**Affiliations:** 1 Department of Neurosurgery, Nanfang Hospital, Southern Medical University, 510515 Guangzhou, Guangdong People’s Republic of China; 2 Department of Neurosurgery, The First Affiliated Hospital of Gannan Medical University, 341000 Ganzhou, Jiangxi People’s Republic of China; 3 Department of Neurosurgery, Shanghai Tenth People’s Hospital, Tongji University School of Medicine, 200072 Shanghai, People’s Republic of China; 4 Department of Neurosurgery, The First Hospital of Quanzhou Affiliated to Fujian Medical University, 362000 Quanzhou, Fujian People’s Republic of China; 5 Department of Neurosurgery, Shenzhen Longgang Central Hospital (The Second Affiliated Hospital of the Chinese University of Hong Kong ((Shenzhen)), Shenzhen, 518116 Guangdong People’s Republic of China

**Keywords:** miR-4310, SP1, PTEN, PI3K/AKT signaling, Glioma

## Abstract

**Background:**

miRNAs have been reported to be involved in multiple biological processes of gliomas. Here, we aimed to analyze miR-4310 and its correlation genes involved in the progression of human glioma.

**Methods:**

miR-4310 expression levels were examined in glioma and non-tumor brain (NB) tissues. The molecular mechanisms of miR-4310 expression and its effects on cell proliferation, migration, and invasion were explored using 3-[4,5-dimethylthiazol-2-yl]-2,5 diphenyl tetrazolium bromide, Transwell chamber, Boyden chamber, and western blot analyses, as well as its effect on tumorigenesis was explored in vivo in nude mice. The relationships between miR-4310, SP1, phosphatase, and tensin homolog (PTEN) were explored using chromatin immunoprecipitation, agarose gel electrophoresis, electrophoresis mobility shift, and dual-luciferase reporter gene assays.

**Results:**

miR-4310 expression was upregulated in glioma tissues compared to that in NB tissues. Overexpressed miR-4310 promoted glioma cell proliferation, migration, and invasion in vitro, as well as tumorigenesis in vivo. The inhibition of miR-4310 expression was sufficient to reverse these results. Mechanistic analyses revealed that miR-4310 promoted glioma progression through the PI3K/AKT pathway by targeting PTEN. Additionally, SP1 induced the expression of miR-4310 by binding to its promoter region.

**Conclusion:**

miR-4310 promotes the progression of glioma by targeting PTEN and activating the PI3K/AKT pathway; meanwhile, the expression of miR-4310 was induced by SP1.

## Background

Glioma is the most common primary intraparenchymal central nervous system (CNS) tumor. Brain tumors are classified according to the World Health Organization (WHO) CNS tumor grading system. In the revised 2016 WHO classification of CNS tumors, numerous molecular markers (IDH, 1p/19q codeletion, H3 Lys27Met, and RELA-fusion) are used in combination with histology for pathological diagnosis [1–4]. Although we now have a more accurate diagnosis of glioma, the prognosis for patients with malignant glioma remains very poor since less than 5% of them have a 5 year relative survival [5].

PTEN is a common tumor suppressor gene. The function of PTEN mainly involves the regulation of the PI3K/AKT pathway. PTEN antagonizes the function of PI3K by dephosphorylating PIP3 to PIP2, thereby regulating the effect of the AKT downstream pathway. PTEN negatively regulates the PI3K/AKT pathway to mediate cell reproduction, invasion, and migration [6–8]. Studies have shown that PTEN is frequently mutated in various cancers, including gliomas. The frequency of PTEN loss of heterozygosity (LOH) in human high-grade gliomas can be up to 70% [8, 9]. PTEN plays important roles in the regulation of cell proliferation, apoptosis, and tumor invasion. Clinical findings in high-grade gliomas suggest that PTEN gene alterations are associated with poor prognosis and may influence the response to specific therapies [10, 11]. One of the mechanisms involved in the regulation of PTEN dosage occurs through micro-RNAs (miRNAs) [12, 13].

miRNAs are short, approximately 20–24 nucleotide (nt), non-coding RNAs that are involved in post-transcriptional regulation of gene expression in multicellular organisms by affecting both the stability and translation of mRNAs. Several studies have reported that miRNAs are closely associated with cancer, including gliomas [14–21]. miR-4310 is a newly discovered miRNA that has been reported to be associated with colon cancer [22].

In our study, we performed numerous experiments, aiming to validate the hypothesis that miR-4310 can promote the proliferation, migration, and invasion of glioma cells. Additionally, we intended to elucidate the mechanism underlying the functions of miR-4310; that is, how miR-4310 promotes the activation of the PI3K/AKT pathway by targeting PTEN. Our results indicated that SP1 regulates the expression of miR-4310 by binding to the promoter region of miR-4310. These findings elucidate the molecular mechanisms controlling glioma progression and contribute to the use of molecular screening markers for targeted therapeutic intervention of glioma.

## Methods

### Cell culture and sample collection

The human glioma cell line LN229, U87 were purchased from the Chinese Academy of Sciences (Shanghai, China) and grown in Dulbecco’s modified Eagle’s medium (DMEM) supplemented with 10% fetal calf serum (Biowest). All cell lines were cultured at 37 °C in a humidified atmosphere of 5% CO2.

A total of 29 glioma tissues and 9 (non-tumor brain) NB tissues samples were obtained from the Nanfang Hospital of Southern Medical University, Guangzhou, China. For the use of these clinical materials for research purposes, prior consent from patients and approval from the Ethics Committees of Nanfang Hospital were obtained. All specimens had confirmed pathological diagnosis and were classified according to the World Health Organization (WHO) criteria.

### Cell transfection

Plasmids were purchased from Vigene Biosciences (Shangdong, China). siRNAs, mimics and inhibitors were designed and synthesized by Guangzhou RiboBio Co., Ltd. (Guangzhou, China) (Additional file 1: Table S1). Exponentially growing cells were seeded in a cell culture plate or dish (NEST Biotech Co., Ltd., China) before transfection. Plasmids, mimics and inhibitors were then transfected into cells using Lipofectamine TM 2000 (Invitrogen Biotechnology Co., Ltd., Shanghai, China) according to the manufacturer’s protocol. Cells were collected 48–72 h after transfection for further experiments.

### Lentivirus production and infection

Lentiviral particles encoding hsa-miR-4310 were designed and constructed by GeneChem (Shanghai, China). Cells were infected with lentiviral vector, and the expression of miR-4310 was detected by qPCR.

### RNA isolation, reverse transcription, and qPCR

Total RNA was isolated from cells or harvested tissues. cDNA was synthesized using reverse transcription reagents (TaKaRa Bio, Inc., Shiga, Japan), and cDNA was used as a template for amplification using specific primers. The Bio-Rad T100 and Bio-Rad CFX96 detection systems were applied for RT-PCR and QPCR, respectively, according to the manufacturer’s instructions. Related primers are shown in Additional file 1: Table S1.

### Western blot analysis

Cell lysates were obtained in lysis buffer, and protein concentrations were determined using a BCA protein assay kit (Thermo Scientific, Waltham, MA, USA). Proteins were separated by SDS-PAGE and transferred onto polyvinyl difluoride membranes, which were immunoprobed with the corresponding antibodies. The proteins were detected using enhanced chemiluminescence reagent (Millipore, USA). Antibodies against the following proteins were used: ZEB1, N-Cadherin, E- Cadherin, PI3K, p-PI3K, AKT, p-AKT, PTEN, p21, p27, GAPDH, β-actin. Images were captured using a ChemiDocTM CRS + Molecular Imager (Bio-Rad, Hercules, CA, USA). Antibody information and dilution are shown in Additional file 1: Table S2. Protein quantitative analysis result are shown in Additional file 2: Fig. S1. 

### Migration and invasion assay

The transwell and boyden assay was used to test cell migration and invasion abilities. Cells were suspended in 100 mL DMEM without serum and seeded into the top chamber of the transwells coated with Matrigel (BD Biosciences, NJ, USA) or left uncoated, and the bottom chambers were filled with 500 mL DMEM supplemented with 10% FBS. The migrated cells were stained with crystal violet and then photographed and quantified by counting the cell numbers in five random fields. All assays were independently performed in triplicate.

### Wound healing assay

Cells were seeded and grew in 6-well plates until a confluent monolayer was reached, and scratches (wounding) were created using a 10 μL pipette tip. Progression of migration was photographed at initiation and 12 h after wounding. All experiments were repeated at least three times.

### MTT assay

Cell proliferation were determined using the MTT assay. Cells were seeded into 96-well plates at a density of 1000 cells/well and incubated overnight to allow cell adherence. Cell viability was measured using MTT (5 mg/mL) (Sigma-Aldrich, MO, USA). The absorbance value (OD) of each well was measured at 490 nm.

### EdU incorporation assay

EdU incorporation was assessed using an Apollo567 In Vitro Imaging Kit (RiboBio Co., Ltd., Guangzhou, China) according to the manufacturer’s protocol. Cells were incubated with 10 μM EdU for 2 h and then fixed with 4% paraformaldehyde. After permeabilization with 0.3% Triton X-100, the cells were stained with Apollo fluorescent dyes and the cell nuclei were stained with 5 μg/mL DAPI. All assays were independently performed in triplicate.

### Electrophoretic mobility shift assay (EMSA)

An electrophoretic mobility shift assay was conducted using an EMSA Kit (BersinBio, Guangzhou, China) according to the manufacturer's instructions. Nuclear extracts were obtained from cells, and their concentrations were determined using a BCA assay kit. An EMSA was performed with a reaction mixture containing nuclear extracts and biotin-labeled probes. Competition or supershift assays were performed by adding a 100-fold excess of cold competitors (unlabeled wild-type or mutant probes) or polyclonal rabbit anti-SP1 (Cell Signaling Technology) to the reaction mixture. After electrophoresis and incubation, signals were recorded and analyzed. Related sequences are shown in Additional file 1: Table S1.

### Luciferase reporter assays

A fragment of the PTEN 3′-UTR (wild-type 3′-UTR) was amplified. Site-directed mutagenesis (mut) of the miR-4310-binding site or miR-4310 promoter region binding site was conducted using the GeneTailor Site-Directed Mutagenesis System (Invitrogen, Guangzhou, China). The wt 3′-UTR or mut 3′-UTR were cloned into the pENTER vector for luciferase reporter assays. The vector was cotransfected with miR-4310 mimics/inhibitor or the control sequence into cells, and luciferase activity was measured 48 h after transfection using the Dual-Luciferase Reporter Assay System (Promega Corporation, Madison, WI, USA).

To investigate the effect of SP1 on the transcriptional activity of miR-4310, fragments encoding SP1-binding sites were cloned into the pGL4.1-Basic luciferase reporter vector, and vectors containing mutant SP1-binding sites were also constructed. These vectors and the SP1 plasmid were cotransfected into cells, following which luciferase activity was detected. Related sequences are shown in Additional file 1: Table S1.

### In situ hybridization (ISH) and evaluation of ISH staining

Tissue sections were deparaffinized in xylene and rehydrated in a graded alcohol series and distilled water. After treatment with proteinase K at 37 °C for 30 min, the sections were rinsed, fixed and then prehybridized for 2 h. Hybridization was performed with miRCURY miR-4310 digoxygenin-labeled probes designed and synthesized by BersinBio (Guangzhou, China). The tissue sections were then washed and incubated with anti-digoxygenin-HRP Fab fragments for 1 h at room temperature. Positive miR-4310 staining was observed by adding BM purple alkaline phosphatase substrate (Roche, Basel, Switzerland) according to the manufacturer’s instructions.

The intensity of staining was scored on a scale of 0 to 3, in which 0 = negative staining, 1 = weakly positive staining, 2 = moderately positive staining, and 3 = strongly positive. The percentage of staining was estimated on a scale of 0 to 4, in which 0 = none, 1 = positive staining in 1–25% of cancer cells, 2 = positive staining in 26 –50%; 3 = positive staining in 51–75%; and 4 = positive staining in 76–100%. The immunohistochemical score (IS) was calculated through multiplying the intensity score by the percentage score. Samples with IS between 0 and 1 were classified as Score 0, samples with IS between 2 and 4 were Score 1, samples with IS between 5 and 8 were Score 2, and samples with IS between 9 and 12 were Score 3 [23]. Then a score of 0–1 is considered as low expression, and a score of 2–3 is considered as high expression.

### Immunohistochemistry (IHC) and evaluation of immunohistochemical staining

Paraffinized sample sections were deparaffinized and dehydrated, and antigen retrieval was then performed in citrate buffer for 3 min. Endogenous peroxidase activity and nonspecific antigens were blocked with 3% H2O2 and goat serum followed by incubation with antibodies overnight at 4 °C. After washing, the sections were incubated with HRP-conjugated secondary antibody and visualized using DAB substrate (Maixin Biotech. Co., Ltd., Fuzhou, China). The evaluation of immunohistochemical staining was scored as ISH staining.

### Animal studies

Animal experimental protocols were approved by The Institutional Animal Ethical Committee, Experimental Animal Center of Southern Medical University, China.

The subcutaneous xenograft mouse model was adopted to evaluate tumor growth, in which 5 × 10^6^ cells in 0.1 mL PBS medium were injected into the left–right symmetric flank of 3–4 week-old male BALB/c nu/nu mice. Mice were sacrificed 30 days after cell inoculation, and tumors were excised, weighed, and processed for further experimentation. Tumor size was determined using measurements of the shortest diameter (A) and the longest diameter (B) with a caliper. The volume was calculated using the formula V = (A^2^ × B)/2.

### Statistical analysis

All data were analyzed using SPSS 20.0 (SPSS, Inc., Chicago, IL, USA) and Graph Pad Prism 6.0. The data are presented as the means ± SDs. Statistical significance was detected using Student’s two-tailed *t*-test for differences between two groups, one-way ANOVA for differences between multiple groups, the general linear model repeated measures variance analysis for differences in tumor growth and MTT assay results. Correlations between gene expression and clinicopathological characteristics were assessed by the chi-square test. Cox regression analysis and Kaplan–Meier survival analysis were used for analyzing the relationship between the variables and patient’s survival time.

All statistical tests were two-sided, and a *P* value of < 0.05 indicated statistical significance (*P < 0.05, **P < 0.01, ***P < 0.001 and ****P < 0.0001).

## Results

### miR-4310 promotes glioma cell proliferation, migration, and invasion in vitro

**Fig. 1:**
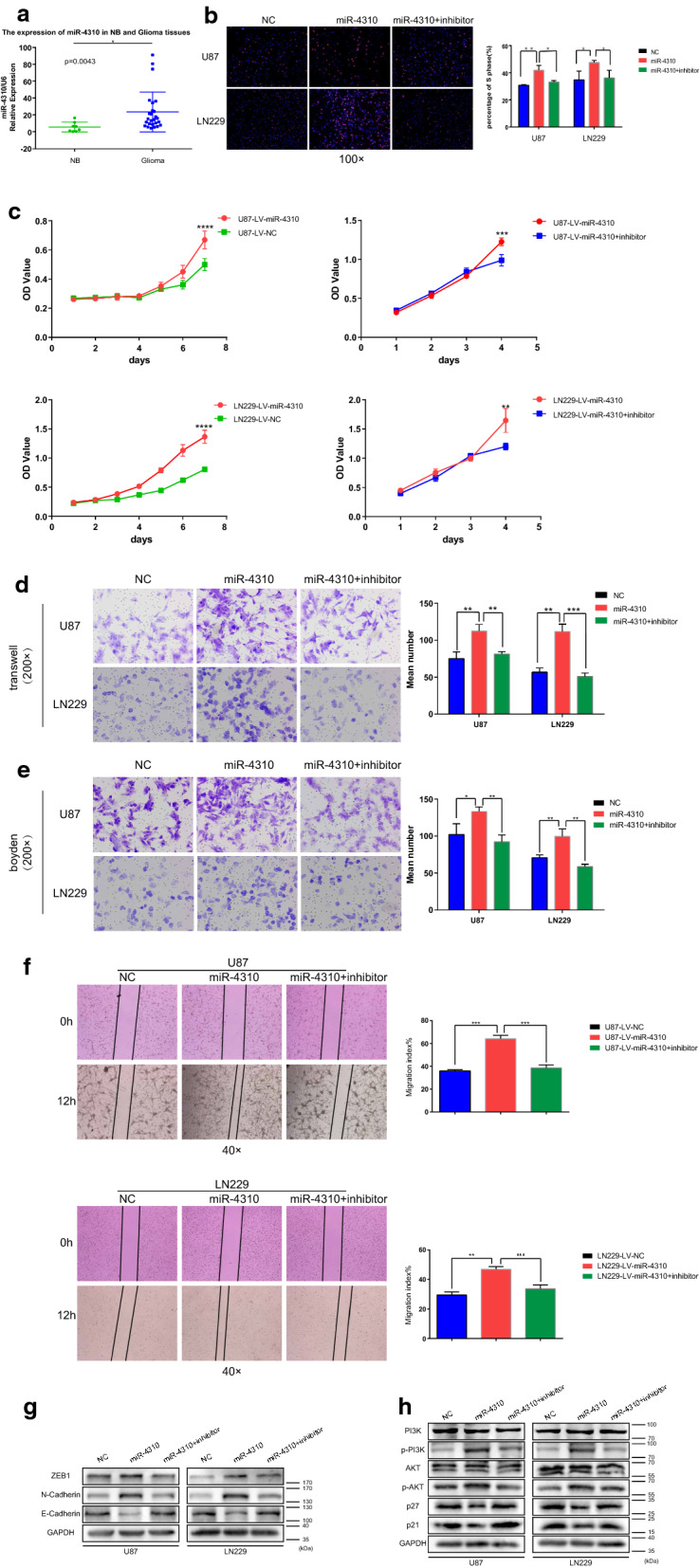
miR-4310 promotes glioma cell proliferation, migration, and invasion by activating PI3K/AKT pathway and promoting EMT progress. **a** The expression of miR-4310 in NB tissues and glioma tissues were examined by qPCR. **b** The proliferative ability of indicated U87 and LN229 cells were evaluated by EdU assays; **c **The cell viability of indicated U87 and LN229 cells were evaluated by MTT assays. **d**–**f** The migration ability and invasion ability of U87 and LN229 cells were tested by Transwell assays (**d**), Boyden assays (**e**) and wound healing assay (**f**). **g**, **h** The expression of PI3K/AKT pathway and EMT related proteins were examined by Western blot. GAPDH were used as a loading control. Data were presented as mean ± s.d. NS, no statistical significance, *p<0.05, **p < 0.01, ***p < 0.001, ****p < 0.0001

To explore the roles of miR-4310 in glioma progression, we first compared the expression of miR-4310 between 8 NB tissues and 26 glioma tissues. We found that glioma tissues showed higher levels of miR-4310 than NB tissues (Fig. 1a).

To further verify the biological function of miR-4310 in glioma cells, we transfected the U87 and LN229 glioma cell lines with the miR-4310 lentivirus and its negative control (NC). We thus obtained two groups of glioma cell lines that could stably express miR-4310 and their respective negative control cell lines. We named them U87-LV-NC and U87-LV-miR-4310, and LN229-LV-NC and LN229-LV-miR-4310 and confirmed high miR-4310 overexpression efficiency in these lines using qPCR (Additional file 3: Fig. S2a, b). This allowed us to use them as tool cell lines in our subsequent studies.

First, we studied the in vitro effects of miR-4310 expression on cell proliferation. For this purpose, we applied Edu incorporation and MTT assays on our U87 and LN229 glioma cell lines. Our results revealed that the overexpression of miR-4310 promoted cell proliferation significantly, whereas the suppression of miR-4310 expression restored the proliferation rate (Fig. 1b, c).

Next, we performed the Transwell chamber, Boyden chamber, and wound healing assays, which showed that overexpressed miR-4310 could promote glioma cell migration and invasion, and this function could be restored by miR-4310 inhibition (Fig. 1d–f).

In conclusion, our in vitro results showed that miR-4310 could indeed promote the proliferation, migration, and invasion of glioma cell lines.

### miR-4310 promotes tumorigenesis in vivo

**Fig. 2:**
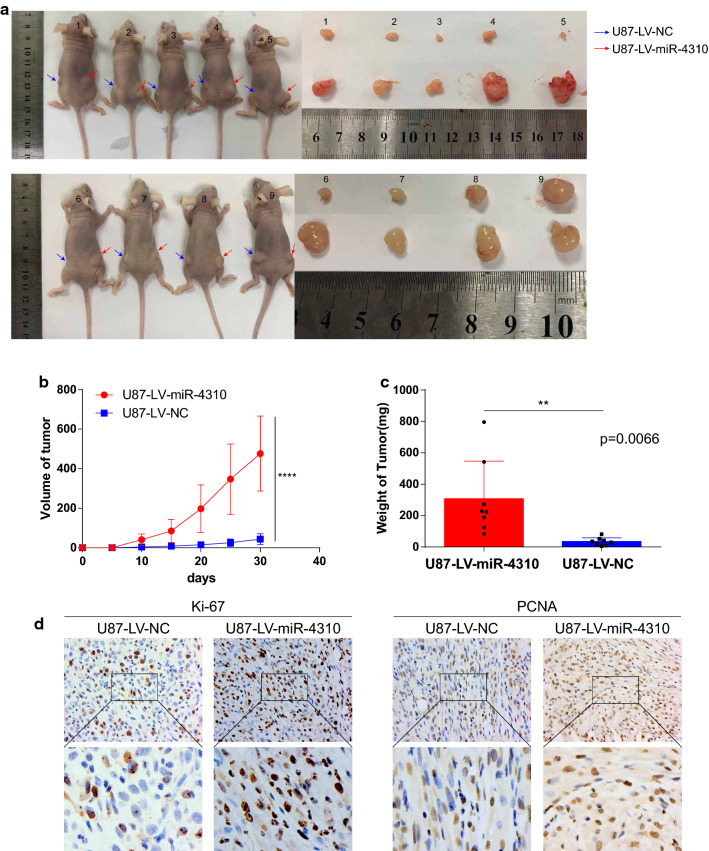
miR-4310 promotes tumorigenesis in vivo. **a** Images of xenograft tumor models injected with U87 cells transfected with miR-4310 lentiviral expression particles or negative control (NC). **b** Tumor volume was measured every five days for each mouse and tumor growth curve was plotted. **c** Xenograft tumors from miR-4310 group and control group were weighed at day 30. **d** Expression of ki-67 and PCNA were detected in xenograft tumors from mice. Data were presented as mean ± s.d. *NS* no statistical significance, *p<0.05, **p < 0.01, ***p < 0.001, ****p < 0.0001

The above results prompted us to perform an in vivo tumor formation experiment by subcutaneously injecting U87-miR-4310 or control cells into nude mice. For this purpose, subcutaneous tumor formation was attempted in 10 nude mice, of which tumorigenesis was successfully achieved in 9, and 1 died halfway. After 30 days of implantation, 8 out of the 9 mice injected with U87-miR-4310 cells had larger tumor burdens (Fig. 2a) and displayed higher expression of Ki67 and proliferating cell nuclear antigen (PCNA) in tumor tissues relative to controls (Fig. 2b). These results suggested that miR-4310 significantly promoted tumorigenesis in vivo.

### Biological function of miR-4310 is achieved by activating the PI3K/AKT pathway and EMT-associated genes

The epithelial-mesenchymal transition (EMT) process and the PI3K/AKT signaling pathway are known to have an inseparable relationship with tumor cell proliferation, migration, and invasion. Thus, we sought to investigate the expression levels of proteins related to EMT and the PI3K/AKT pathway by western blot. Our results showed that overexpression of miR-4310 upregulated the zinc finger E-box binding homeobox 1 (ZEB1) and N-cadherin, and downregulated E-cadherin (Fig. 1g). We then examined the effect of miR-4310 on the PI3K/AKT pathway. We found that overexpression of miR-4310 significantly increased the phosphorylation of PI3K and AKT, but not their total protein levels and downregulated the downstream molecules of PI3K/AKT, p21, and p27 (Fig. 1h). All the above effects were restored in the presence of the miR-4310 inhibitor.

The above experimental results show that miR-4310 achieves its biological functions by activating the EMT and the PI3K/AKT signaling pathway.

### miR-4310 directly targets PTEN

The biological function of miRNAs is achieved by binding to their target gene mRNA. PTEN is an important factor that antagonizes the PI3K/AKT signaling pathway. Therefore, we supposed that PTEN might be the target gene of miR-4310. miR-4310 could release the antagonistic effect of PTEN on the PI3K/AKT pathway by targeting PTEN, thereby activating this pathway.

**Fig. 3:**
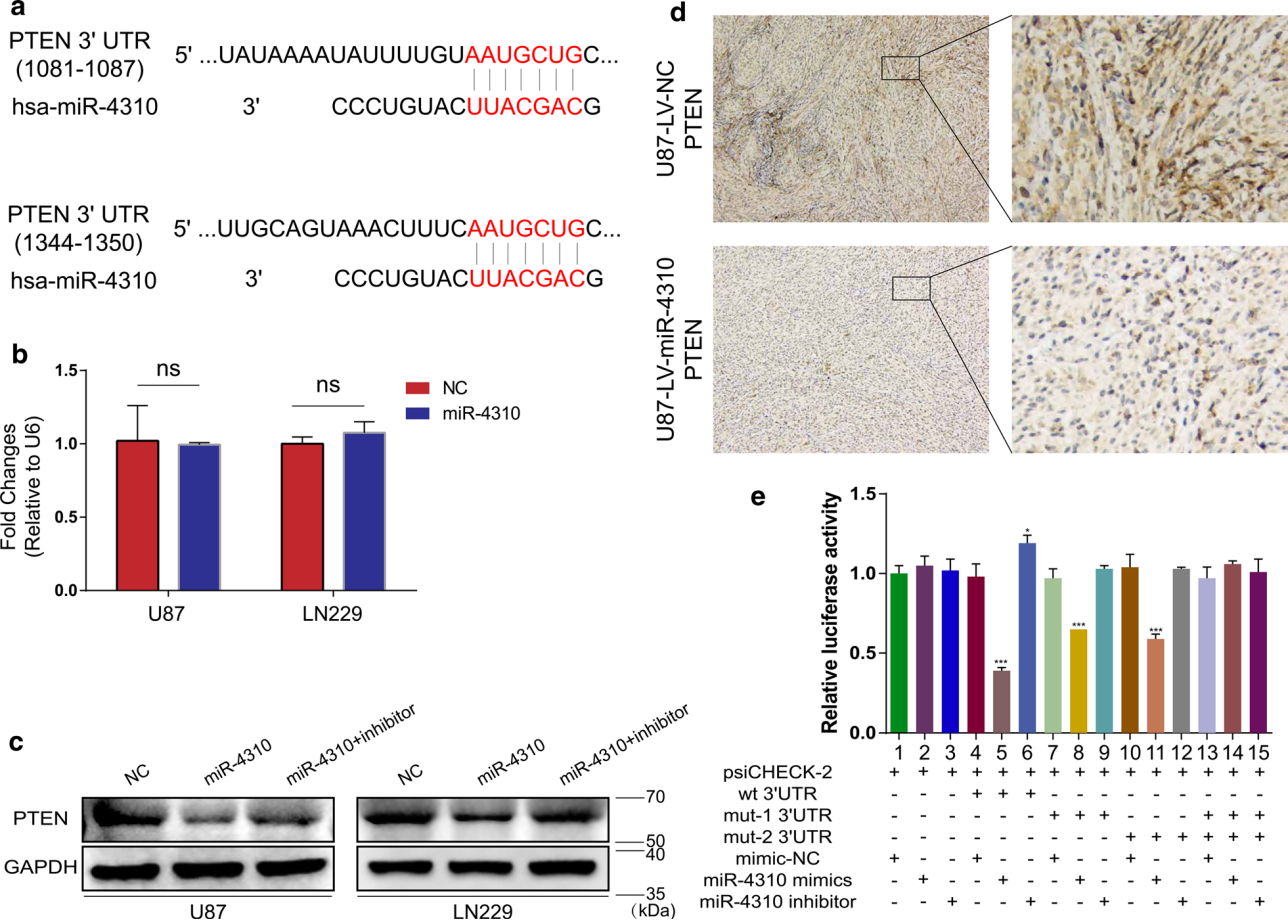
miR-4310 directly targets PTEN. **a** Schematic diagram of putative binding sequences of miR-4310 in the 3′-UTR of PTEN. **b** PTEN mRNA expression was detected by qPCR in miR-4310-overexpressing U87 and LN229 cells. **c** PTEN protein level was detected in U87 and LN229 cells under LV-miR-4310 transfection or co-transfection with miR-4310 inhibitor. **d** PTEN expression was evaluated by IHC staining in xenografts derived from U87-LV-NC and U87-LV-miR-4310 cells. **e** Luciferase reporter assay was used to determine the miR-4310 binding sites in the PTEN 3′-UTR. Data were presented as mean ± s.d. *NS* no statistical significance, *p<0.05, **p < 0.01, ***p < 0.001, ****p < 0.0001

We used TargetScan (http://www.targetscan.org/) and predicted two PTEN sites that could be direct targets of miR-4310 (Fig. 3a). Overexpression of miR-4310 downregulated the PTEN protein levels but did not affect the mRNA level (Fig. 3b, c). This indicates that miR-4310 can regulate the translation of the PTEN mRNA. This result was confirmed by immunohistochemistry staining in xenografts derived from U87-LV-NC and U87-LV-miR-4310 cells (Fig. 3d).

To further prove that miR-4310 directly targets PTEN, we performed the dual-luciferase reporter gene assay. Our results showed that the luciferase activity was abrogated when co-transfected with wild-type PTEN reporter and miR-4310 mimics (Fig. 3e lane 5). This result could be reversed by co-transfection with wild-type PTEN reporter and miR-4310 inhibitor (Fig. 3e lane 6) but was not affected when co-transfected with mutant PTEN reporter and miR-4310 mimics or inhibitor (Fig. 3e lane 14, 15). Moreover, when we singly co-transfected with mutant site 1 or site 2 of PTEN reporter and miR-4310 mimics, we could still observe that luciferase activity was abrogated (Fig. 3e lane 8, 11), but the degree of abrogation was less than that of lane 5 (Fig. 3e). Thus, the results of the dual-luciferase reporter gene assay illustrate that PTEN is the target gene of miR-4310, and both binding sites are active.

**Fig. 4:**
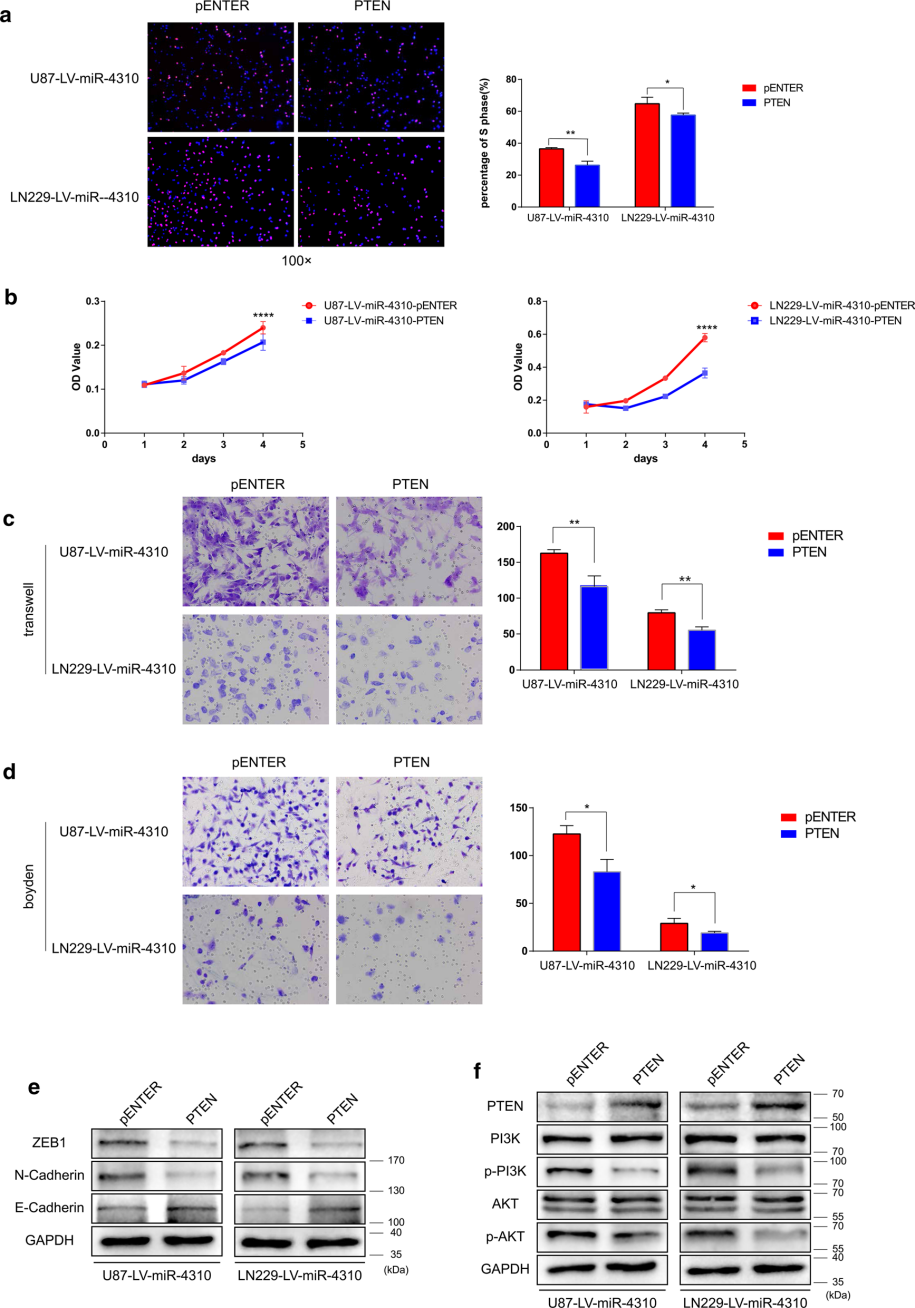
Overexpression of PTEN can reverse the biological functions mediated by miR-4310. **a**, **b** Proliferative ability of U87 and LN229 cells co-transfected with PTEN plasmid and LV-miR-4310 was examined by EdU assays (**a**) and MTT assays (**b**). **c**, **d** Migration and invasion of U87 and LN229 cells co-transfected with PTEN plasmid and LV-miR-4310 were examined by Transwell assays (**c**) and Boyden assays (**d**), respectively. **e**, **f** Expression of ZEB1, N -Cadherin, E-Cadherin, PI3K, AKT, p-PI3K and p-AKT were detected by western blot in U87-LV-miR-4310 and LN229-LV-miR-4310 performed after transfection with pENTER plasmid (the control group) and PTEN plasmid (the treatment group) as indicated. GAPDH were used as a loading control. Data were presented as mean ± s.d. *NS* no statistical significance, *p<0.05, **p < 0.01, ***p < 0.001, ****p < 0.0001

To understand whether the biological function of miR-4310 is achieved through PTEN, we transiently transfected a PTEN plasmid into the U87-LV-miR-4310 and LN229-LV-miR-4310 cell lines to test whether overexpression of PTEN could inhibit the proliferation, migration, and invasion of these cells. The obtained results showed that the overexpression of PTEN could inhibit U87-LV-miR-4310 and LN229-LV-miR-4310 proliferation, migration, and invasion (Fig. 4a–d). The immunoblotting assay data also confirmed that the overexpression of PTEN could affect the EMT and PI3K/AKT associated proteins. (Fig. 4e, f).

Collectively, these results support our initial hypothesis that miR-4310 promotes the activation of the PI3K/AKT pathway by targeting PTEN, thus causing the release of the antagonistic effect of PTEN on the signaling pathway.

### SP1 induces the expression of miR-4310 by binding to its promoter region

SP1 is a zinc finger transcription factor that binds to GC-rich motifs of many promoters. The encoded protein is involved in many cellular processes, including cell differentiation, cell growth, apoptosis, immune responses, response to DNA damage, and chromatin remodeling. According to reports, SP1 plays an important role in the tumorigenesis, progression, and drug resistance of gliomas [24–27]. We found that the promoter region of miR-4310 also contains GC-rich fragments. Thus, we supposed that SP1 may be an upstream signaling molecule of miR-4310 and may regulate miR-4310 expression by binding to the promoter region of miR-4310.

**Fig. 5:**
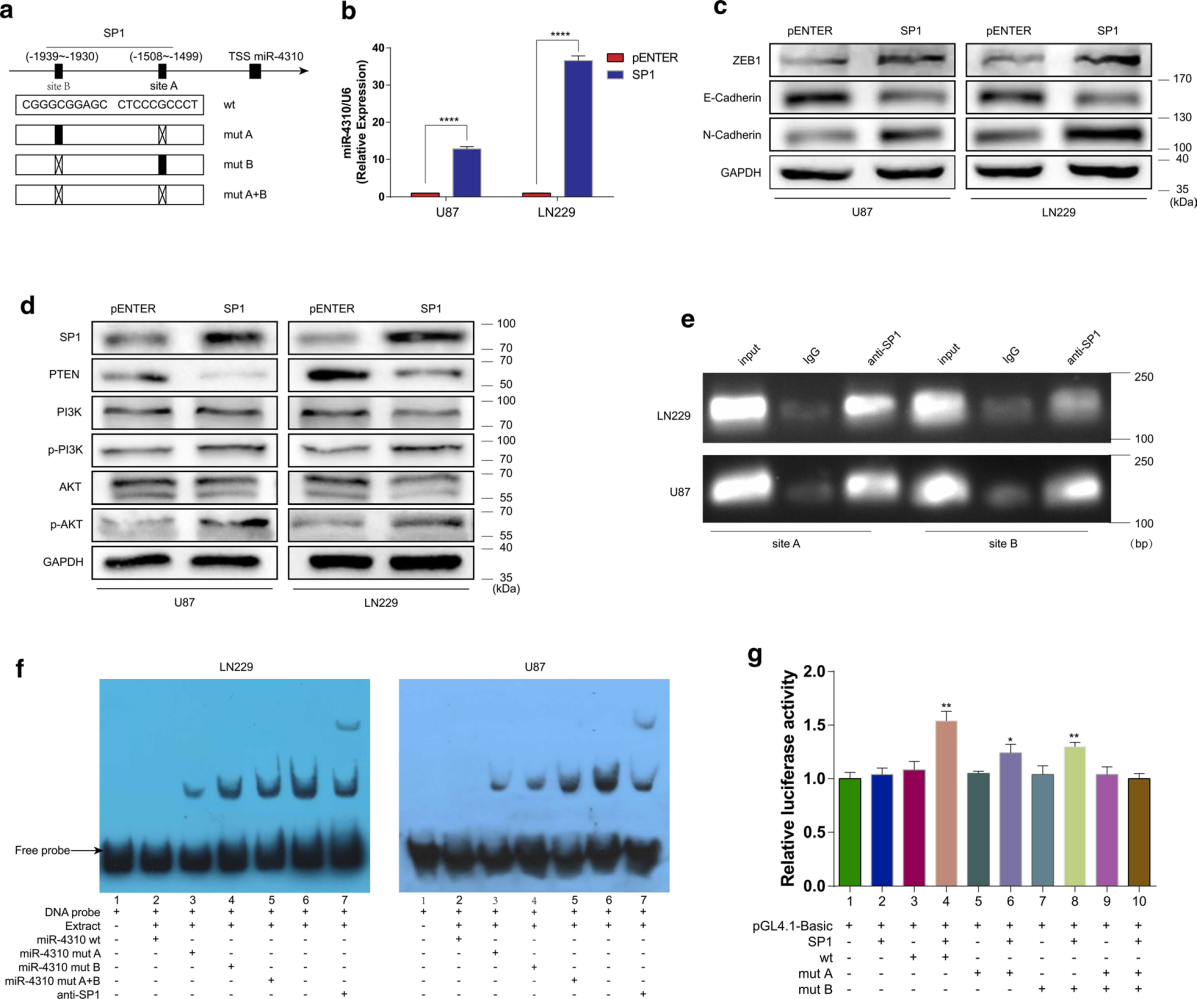
SP1 induces the expression of miR-4310 by binding to its promoter region. **a** Schematic diagram of the promoter regions of miR-4310 with the putative SP1 TFBSs (site A and site B) and the structure of the wild-type (WT) and TFBS mutant (mut A, mut B, and mut A + B) luciferase reporters driven by the promoter. **b** Expression of SP1 was detected by qPCR assays. **c**, **d** The expression of PI3K/AKT pathway and EMT related proteins were examined by Western blot. GAPDH were used as a loading control. **e** ChIP assay along with PCR and agarose gel electrophoresis showed amplification of SP1-binding sites A and B. **f** Protein-DNA interactions between SP1 and the miR-4310 promoter were determined by electrophoretic mobility shift assays (EMSA). **g** Luciferase reporter assay was used to determine the binding of SP1 to miR-4310 promoter region. Statistical methods: One-way ANOVA and Dunnett’s multiple comparison test. Data were presented as mean ± s.d. *NS* no statistical significance, *p<0.05, **p < 0.01, ***p < 0.001, ****p < 0.0001

We used UCSC (http://genome.ucsc.edu), PROMO (http://alggen.lsi.upc.es/), and JASPA (http://jaspar.genereg.net/) to predict whether SP1 could bind to certain sequences in the promoter region of miR-4310. Two sites were identified that SP1 could bind to: site A (− 1508– − 1499) and site B (− 1939–− 1930) (Fig. 5a). We overexpressed SP1 in U87 and LN229 glioma cell lines and used PCR to detect the level of miR-4310. The results showed that the expression level of miR-4310 increased (Fig. 5b). Similarly, the PTEN/PI3K/AKT pathway and EMT related proteins showed the same regulation. (Fig. 5c, d) This proves that SP1 may act as an upstream factor of miR-4310 to regulate the expression of miR-4310.

We then verified our hypothesis using chromatin immunoprecipitation (ChIP) and agarose gel electrophoresis assay on U87 and LN229 cells. Site A and site B sequences were enriched in the anti-SP1 group, indicating that SP1 can bind to site A and site B (Fig. 5e). Subsequently, we used electrophoresis mobility shift assay (EMSA) and dual-luciferase reporter gene assay to further confirm this conclusion (Fig. 5f, g). These results also supported that SP1 could induce the expression of miR-4310 by binding to its promoter region.

### Clinical relationship among miR-4310, SP1, and PTEN

**Fig. 6:**
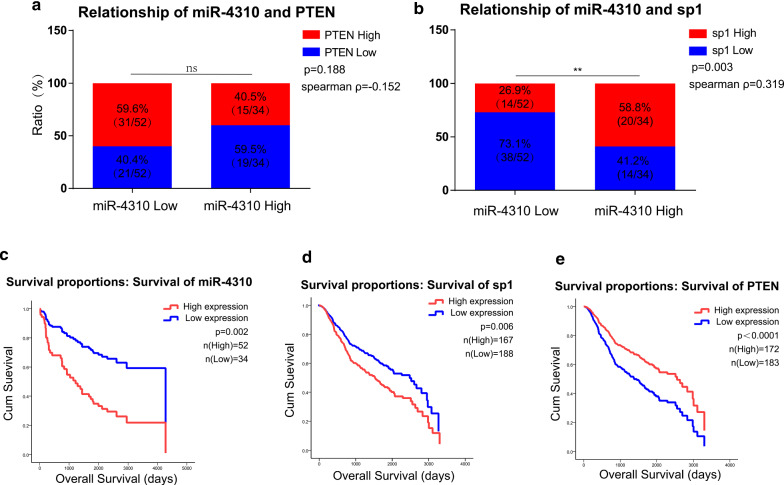
Clinical relationship among miR-4310, SP1, and PTEN. **a**, **b** Correlations between miR-4310 and PTEN, SP1 and miR-4310 expression level scored by ISH or IHC staining were shown. **c**–e Cox regression analysis of miR-4310, PTEN and SP1 expression in glioma cohorts from Nanfang Hospital neurosurgery department (**c**), CGGA-693 database (**d**, **e**). Data were presented as mean ± s.d.. NS, no statistical significance, *p<0.05, **p < 0.01, ***p < 0.001, ****p < 0.0001

To further clarify the relationship among miR-4310, PTEN, and SP1, we used in situ hybridization (ISH) and immunohistochemistry (IHC) to semi-quantitatively analyze their expression levels in 86 paraffin-embedded glioma tissue samples. Among the 52 samples with low expression levels of miR-4310, 59.6% (31/ 52) also exhibited high expression of PTEN and 40.4% (21/52) exhibited low expression of PTEN, whereas 26.9% (14/52) exhibited high expression of SP1 and 79.1% (38/52) exhibited low expression of SP1. Similarly, of the 34 samples with high expression levels of miR-4310, 40.5% (15/34) presented with high expression of PTEN and 59.5% (19/34) with low expression of PTEN, whereas 58.8% (20/34) presented with high expression of SP1 and 41.2% (14/34) with low expression of SP1 (Fig. 6a, b). Our results showed that SP1 positively correlated with miR-4310 (Chi-square test, *p* = 0.003; Spearman correlation coefficient ρ = 0.319). Maybe due to the small size of our sample, we were not able to find a significant negative correlation between miR-4310 and PTEN, but they still had a negative tendency to some extent (Chi-square test, *p* = 0.188; Spearman correlation coefficient ρ = − 0.152). The other clinical characteristics of glioma patients are summarized in Table 1.

**Table 1.** Correlation between the other characteristics and miR-4310 expression in glioma

Characteristics		n	miR-4310 expression		*p* value
			High	Low	
	0–20	13	5	8	0.393
Age(years)	20–50	57	25	32	
	50–80	16	4	12	
Gender	Male	50	16	34	0.119
	Female	36	18	18	
Grade	WHO I-II	52	29	23	0.079
	WHO III-IV	34	12	22	

### miR-4310, SP1, and PTEN are independent prognostic factors for glioma

According to CGGA (http://www.cgga.org.cn), we analyzed the expression levels of SP1 and PTEN in each glioma grade and performed Kaplan–Meier survival analysis based on SP1 and PTEN expressions. The results of this analysis show that the expression level of SP1 increases with the WHO grade of glioma (Additional file 3: Fig. S2c, e). Moreover, the Kaplan–Meier survival analysis showed that high expression of SP1 is associated with poor prognosis in glioma patients (Additional file 3: Fig. S2d, f). Similarly, high expression of PTEN is associated with a good prognosis in glioma patients (Additional file 3: Fig. S2g, h). We still performed a Kaplan–Meier survival analysis on 86 glioma patients. The result showed that patients with high SP1 expression had poorer OS rates than those exhibiting low SP1 expression (Additional file 3: Fig. S2i).

Although neither *p* value reached significance, in these tissue samples, patients with high miR-4310 expression or PTEN expression tended to have longer or shorter survival, respectively (Additional file 3: Fig. S2j, k). We suspect that the non-significance is due to the small sample size and mutual interference among SP1, PTEN, and miR-4310.

Furthermore, we used univariate and multivariate COX regression to analyze whether miR-4310, SP1, and PTEN are independent prognostic factors for glioma. We first used data from these 86 cases to analyze the relationship between various factors and the overall survival (OS) of glioma patients (Table 2). Age, gender, WHO grade, SP1, PTEN, and miR-4310 were included in this study as possible prognostic factors.

**Table 2:** Summary of univariate and multivariate Cox regression analysis of overall survival duration. (Data from Nanfang Hospital neurosurgery department)

	Univariate analysis			Multivariate analysis (Forward:LR)		
	p	HR	95% CI	*p*	HR	95% CI
Age (years)						
0–20	0.1	–	–	0.148	–	–
20–50	0.046	0.309	0.097–0.981	0.854	–	–
50–80	0.107	0.561	0.278–1.133	0.082	–	–
Gender	0.585	0.846	0.464–1.543	0.85	–	–
Grade						
WHO I	<0.0001	–	–	<0.0001		
WHO II	0.034	0.114	0.015–0.852	0.022	0.096	0.013–0.717
WHO III	<0.0001	0.216	0.109–0.427	<0.0001	0.134	0.062–0.289
WHO IV	0.047	0.422	0.180–0.988	0.022	0.359	0.150–0.861
sp1	0.046	0.54	0.295–0.989	0.051	–	–
PTEN	0.134	0.635	0.350–1.150	0.243	–	–
miR-4310	0.227	0.688	0.375–1.262	0.002	0.344	0.173–0.684

**Fig. 7:**
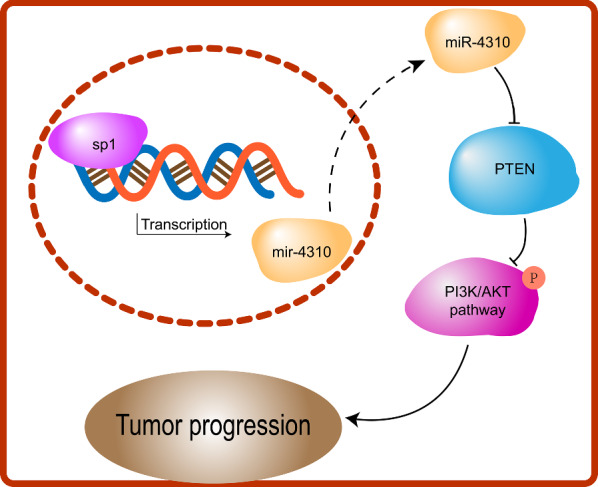
Schematic diagram of SP1/miR-4310/PTEN/PI3K/AKT pathway in glioma. SP1 regulates miR-4310 expression by binding to the miR-4310 promoter region. By targeting PTEN, miR-4310 releases the inhibitory effect of PTEN on the PI3K/AKT pathway and activates the PI3K/AKT pathway, thereby promoting the proliferation, invasion and migration of glioma

The multivariate COX regression results revealed that miR-4310 (*p* = 0.002) and WHO grade (*p* < 0.0001) are prognostic factors for glioma. We next performed a survival analysis based on the miR-4310 expression (Fig. 7d), and these results suggested that high expression of miR-4310 is associated with poor prognosis in glioma patients.

In addition, considering that SP1 and PTEN are also common risk factors clinically, we also used the CGGA-693 database for a further COX regression analysis. Age, gender, WHO grade, SP1, PTEN, IDH mutation, chemotherapy, and radiotherapy were included in the study as possible prognostic factors. The results based on CGGA-693 revealed that WHO grade (*p* < 0.0001), SP1 (*p* = 0.006), PTEN (*p* < 0.0001), IDH mutation (*p* < 0.0001), and chemotherapy (*p* = 0.004) are independent prognostic factors for glioma (Table 3. Survival analysis results suggest that high expression of SP1 is associated with poor prognosis in glioma patients. Similarly, high expression of PTEN correlates with benign prognosis in glioma patients. (Fig. 6d, e).

**Table 3:** Summary of univariate and multivariate Cox regression analysis of overall survival duration. (Data from CGGA-693 database.)

	Univariate analysis			Multivariate analysis (Forward:LR)		
	*p*	HR	95% CI	*p*	HR	95% CI
Age						
0–20	< 0.0001	–	–	0.089	–	–
20–50	0.04	0.229	0.056–0.932	0.029	–	–
50–80	< 0.0001	0.436	0.325–0.584	0.507	–	–
Gender	0.646	0.933	0.696–1.252	0.553	–	–
Grade						
WHO II	< 0.0001	–	–	<0.0001	–	–
WHO III	< 0.0001	0.099	0.063–0.155	<0.0001	0.127	0.074–0.219
WHO IV	< 0.0001	0.292	0.210–0.407	<0.0001	0.431	0.291–0.639
IDH mutant	< 0.0001	4.576	3.350–6.250	<0.0001	2.363	1.627–3.432
Radio status	0.215	0.778	0.523–1.157	0.104	–	–
Chemo status	0.035	0.69	0.489–0.974	0.004	1.787	1.207–2.645
sp1	0.002	0.628	0.466–0.846	0.006	0.643	0.468–0.883
PTEN	0.003	1.561	1.166–2.090	<0.0001	1.734	1.280–2.349

## Discussion

Glioma is a primary brain tumor with a poor prognosis. Despite the recent significant advances in the molecular diagnosis of gliomas, their treatment remains stagnant [28]. Difficulties in the treatment of gliomas are closely related to their invasive growth. Therefore, the study of the aggressive proliferation, migration, and invasion growth behavior of glioma cells is a key measure to overcome the difficulties of glioma treatment.

A growing number of reports demonstrate that miRNAs play an important role in the progression of glioma. More and more miRNAs are found to act as tumor suppressors or oncomiRs in gliomas [29–32]. As research progresses, scientists are beginning to tap the huge potential of miRNAs for disease treatment. For example, anti-miR-122 (Miravirsen) is used for hepatitis C therapy in clinical trials; MRX34 (a synthetic miR-34a mimic loaded in liposomal nanoparticles) is in phase I clinical trial for primary liver cancer and liver metastases [33–38]. Therefore, we believe that miRNAs are potentially beneficial for the development of new glioma therapies.

As a newly discovered miRNA, the role of miR-4310 in glioma has not been reported. Through a series of experiments, we found that miR-4310 significantly promotes the proliferation, migration, and invasion of glioma cells. This suggests that miR-4310 acts as an onco-miRNA in gliomas. miRNAs act by binding to mRNA of the target gene to mediate the degradation of the target gene mRNA or suppress its translation process. It is well established that PTEN is closely associated with tumorigenesis and progression of gliomas [8–11]. Through TargetScan, we predicted that miR-4310 and PTEN have two binding sites. Next, we used dual-luciferase reporter gene experiments to prove that both PTEN sites can bind to miR-4310 and perform its biological function. The role of PTEN and the PI3K/AKT pathway in glioma has been well reported [39–43]. The PI3K/AKT signaling pathway is overactivated in gliomas [44]. AKT is a key factor in this pathway; it regulates cell proliferation, migration, and invasion by regulating important downstream molecules [45–47]. EMT is a feature of tumor cell migration and invasion ability, by which epithelial cells acquire mesenchymal features, lose polarity and tight junctions, and acquire the ability to infiltrate and migrate [48–52]. The PI3K/AKT pathway is closely associated with EMT; hence, in our study, we observed that miR-4310 regulated the proteins related to EMT and PI3K/AKT pathway with the help of western blot analysis. That means the function of miR-4310 is achieved by combining to PTEN to release the inhibitory effect of PTEN on the PI3K/AKT pathway, thereby promoting the progression of glioma.

SP1 is a zinc finger transcription factor that binds to GC-rich motifs of many promoters. Through bioinformatics analysis, we found that the promoter region of miR-4310 also has a GC-rich region. A series of experiments proved that SP1 could induce the expression of miR-4310 by binding to its promoter region. Importantly, we revealed that the SP1/miR-4310/PTEN axis activates the PI3K/AKT signaling pathway to promote glioma progression (Fig. 7).

Finally, we analyzed 86 paraffin-embedded glioma tissue samples and found a positive correlation between miR-4310 and SP1. This is consistent with the conclusions we have provided above. Through Kaplan–Meier survival analysis and Cox regression analysis, we proved that miR-4310, SP1, and PTEN can be used as risk factors for glioma prognosis. Although limited by sample size, ethnicity, geographical restrictions, and other various factors, there are certain contradictions in our conclusions. However, looking at all the data comprehensively, our conclusions remain credible and instructive.

Reportedly, glioma is the most common primary intraparenchymal central nervous system tumor. Patients with glioma have a poor prognosis, especially those with high-grade gliomas. Therefore, exploring the pathogenesis of gliomas and finding new therapeutic approaches are important to overcome the poor prognosis of this disease. In this study, we explored a new regulatory mechanism, as illustrated in our working model in Fig. 7, the SP1/miR-4310/PTEN/PI3K/AKT axis in glioma. This axis expands the molecular regulatory mechanisms in gliomas and improves our understanding of the mechanism of glioma progression. Besides, both SP1 and PTEN play an important role in the tumorigenesis, progression, and drug resistance of gliomas [53–55]. The discovery of new signal regulation pathways will help contribute to the development of new therapies.

## Supplementary information

**Additional file 1: Table S1.** The primers and sequence used in this study. **Table S2.** A list of antibodies used for Western blot, IHC staining, CHIP, EMSA.

**Additional file 2: Figure S1.** **a**–**d** Protein quantitative analysis for Fig. 1g, h. **e** Protein quantitative analysis for Fig. 3c. **f**–**i** Protein quantitative analysis for Fig. 4e, f. **j**–**m** Protein quantitative analysis for Fig. 5c, d.

**Additional file 3: Figure S2.** **a**, **b** Fluorescence analysis and qPCR assay to confirm the efficiency of U87 and LN229 after transfection with lentivirus. **c-h** Expression of SP1 and PTEN in each grade of glioma was shown, and Kaplan-Meier survival analysis based on SP1 and PTEN expression were performed in CGGA datasets. **i-k** Kaplan–Meier survival analysis of overall survival of 86 glioma patients on the basis of SP1, PTEN and miR-4310 expression levels were performed in Nanfang Hospital cohort. **l **The example diagram of high expression and low expression of miR-4310, PTEN and SP1 in ISH or IHC.

## Data Availability

All data generated or analyzed during this study are included in this published article and its supplementary information files.

## Abbreviations

CNS: Central nervous system
WHO: World Health Organization
PTEN: Phosphatase and tensin homolog
LOH: Loss of heterozygosity
MiR-4310: MicroRNA-4310
EMT: Epithelial-mesenchymal transition
NB: Non-tumor brain tissues
NC: Negative control
ZEB1: Zinc finger E-box binding homeobox 1
MTT: 3-[4,5-Dimethylthiazol-2-yl]-2,5 diphenyl tetrazolium bromide
ChIP: Chromatin immunoprecipitation
EMSA: Electrophoresis mobility shift
ISH: In situ hybridization
IHC: Immunohistochemistry
DMEM: Dulbecco’s modified Eagle’s medium
