# Supplementary material for: MiR-4310 induced by SP1 targets PTEN to promote glioma progression
Source: Cancer Cell Int. 2020 Dec 17;20:567. doi: 10.1186/s12935-020-01650-9 (PMC7745362; doi:10.1186/s12935-020-01650-9)
Supplement: Supplementary file 1 — Additional file 1: Table S1. The primers and sequence used in this study. Table S2. A list of antibodies used for Western blot, IHC staining, CHIP, EMSA. [file 12935_2020_1650_MOESM1_ESM.pdf]

Supplementary Table 1: The primers and sequence used in this study.

Gene name		sequence
mir-4310		GCAGCAUUCAUGUCCC
U6		CTCGCTTCGGCAGCACATATA
mir-4310 mimics		GCAGCAUUCAUGUCCC
antisense		GGGACAUGAAUGCUGC
mir-4310 inhibitor		GGGACAUGAAUGCUGC
PTEN	Forward	ACCATAACCCACCACAGCTA
	Reverse	GGAATAGTTACTCCCTTTTTGTCTC
Promoter of mir-4310 site A	Forward	GGAGGAGAGTTCGCAAGAG
	Reverse	TGCTGCCACAGCTTCTC
Promoter of mir-4310 site B	Forward	AGCAGCTCTTCCTCAGAGA
	Reverse	ACTGAGCAGAGAGGAGAATAGA
V80-pmirGLO-PTEN WT		CTCGAGAGCTTCTTTTTTCTCATTAAATATAAAATATTTTGTAATG<br>CTGCACAGAAATTTTCAATTTGAGATTCTACAGTAAGCGTTTTTTT<br>TCTTTGAAGATTTATGATGCACTTATTCAATAGCTGTCAGCCGTTC<br>CACCCTTTTGACCTTACACATTCTATTACAATGAATTTTGCAGTTT<br>TGCACATTTTTTAAATGTCATTAACTGTTAGGGAATTTTACTTGAA<br>TACTGAATACATATAATGTTTATATTAAAAAGGACATTTGTGTTA<br>AAAAGGAAATTAGAGTTGCAGTAAACTTTCAATGCTGCACACAA<br>AAAAAAGACATTTGATTTTTCAGTAGAAATTGTCCTACATGTGCT<br>TTATTGATTTGCTATTGAATCTAGA
V80-pmirGLO-PTEN mut-1		CTCGAGAGCTTCTTTTTTCTCATTAAATATAAAATATTTTGTTTAC<br>GACCACAGAAATTTTCAATTTGAGATTCTACAGTAAGCGTTTTTTT<br>TCTTTGAAGATTTATGATGCACTTATTCAATAGCTGTCAGCCGTTC<br>CACCCTTTTGACCTTACACATTCTATTACAATGAATTTTGCAGTTT<br>TGCACATTTTTTAAATGTCATTAACTGTTAGGGAATTTTACTTGAA<br>TACTGAATACATATAATGTTTATATTAAAAAGGACATTTGTGTTA<br>AAAAGGAAATTAGAGTTGCAGTAAACTTTCAATGCTGCACACAA<br>AAAAAAGACATTTGATTTTTCAGTAGAAATTGTCCTACATGTGCT<br>TTATTGATTTGCTATTGAATCTAGA
V80-pmirGLO-PTEN mut-2		CTCGAGAGCTTCTTTTTTCTCATTAAATATAAAATATTTTGTAATG<br>CTGCACAGAAATTTTCAATTTGAGATTCTACAGTAAGCGTTTTTTT<br>TCTTTGAAGATTTATGATGCACTTATTCAATAGCTGTCAGCCGTTC<br>CACCCTTTTGACCTTACACATTCTATTACAATGAATTTTGCAGTTT<br>TGCACATTTTTTAAATGTCATTAACTGTTAGGGAATTTTACTTGAA<br>TACTGAATACATATAATGTTTATATTAAAAAGGACATTTGTGTTA<br>AAAAGGAAATTAGAGTTGCAGTAAACTTTCTTACGACCACACAA<br>AAAAAAGACATTTGATTTTTCAGTAGAAATTGTCCTACATGTGCT<br>TTATTGATTTGCTATTGAATCTAGA
V80-pmirGLO-PTEN mut-1、2		CTCGAGAGCTTCTTTTTTCTCATTAAATATAAAATATTTTGTTTAC<br>GACCACAGAAATTTTCAATTTGAGATTCTACAGTAAGCGTTTTTTT

	TCTTTGAAGATTTATGATGCACTTATTCAATAGCTGTCAGCCGTTC<br>CACCCTTTTGACCTTACACATTCTATTACAATGAATTTTGCAGTTT<br>TGCACATTTTTTAAATGTCATTAACTGTTAGGGAATTTTACTTGAA<br>TACTGAATACATATAATGTTTATATTAAAAAGGACATTTGTGTTA<br>AAAAGGAAATTAGAGTTGCAGTAAACTTTCTTACGACCACACAA<br>AAAAAAGACATTTGATTTTTCAGTAGAAATTGTCCTACATGTGCT<br>TTATTGATTTGCTATTGAATCTAGA
mir-4310 promoter wild-type probe	AGTGGCTCCGGGCGGAGCTGGAAGGTCCCCTCCCGCCCTCACA
mir-4310 promoter wild-type cold competitor probe	AGTGGCTCCGGGCGGAGCTGGAAGGTCCCCTCCCGCCCTCACA
mir-4310 promoter mutA cold competitor probe	AGTGGCTCGCCCGCCTCGTGGAAGGTCCCCTCCCGCCCTCACA
mir-4310 promoter mutB cold competitor probe	AGTGGCTCCGGGCGGAGCTGGAAGGTCCCGAGGGCGGGACAC<br>A
mir-4310 promoter mut A+B cold competitor probe	AGTGGCTCGCCCGCCTCGTGGAAGGTCCCGAGGGCGGGACACA
mir-4310 promoter wt	GGAGGAGAGTTCGCAAGAGCCTAGCAGTGGCCCGCTGAAGCTC<br>AGTGCCCACCAGTGGCTCCGGGCGGAGCTGGAGGCCCGGGAG<br>AAGCTGTGGCAGCAGGCCACCCAGCTGGGGCAGCAGGCACTTC<br>TTGCTGCAGGGACACCCACCAAGGAGGTGGGCCCCCTCCCCTG<br>GTGCCCCCACATACCCTAGCTGACCAAGCCTGTGCCTGGCCACC<br>TGGTGCCCCGTCCTACTGTGCCATGCTGGCCTCTCCTCAGGAGC<br>TTCCCTCAGGATCCAGTCCTCACCTCACCCACACCACCCTCCCC<br>AGCTGAGGCTCCTCATGTGTCCATCCCCAGGTCCAGGAAGAGCT<br>TCGAGCCCTGCAGGACCAGCGGGACCAGGTGTATCAGACCTGG<br>GCACGGAAGCAAGAGAGGCTGCAGGCCGAGCAGCAGGAGCAG<br>CTCTTCCTCAGAGAGTGCGGCCGCCTGGAGGAGATCCTCGCGG<br>CCCAGGAGGCAGGTCCCCTCCCGCCCTCACACTCCGCTCAGCCT<br>CCTGCTCCTCCTTTCTATTCTCCTCTCTGCTCAGTCCCTTTCCTCCT<br>GCCTCTCTGGCTTTTTCTCTAATATGAGGCAGCTGTGCACAAGTC<br>CTTCCAGGTGGCCATGGTGAAGTGCTGAGGCCCCTGAGCGTCC<br>ACTGACATCCCCAGCCCCTATCCCAGACTGAGCCCCGAGGAAG<br>TTGGCCAGAGGCTGGTCATGCTCCCCACATGGTGCAGAGGTTGA<br>GGTATCGCAGCCTCCCAACCTGGACTCCCACTCCTCACAGCTCC<br>ATGCCAAGCTCAGTGGGCACTCCTAAGGCCCTGCCATACCCAGA<br>GTGCCCATGGGCACAGACTGCAGCTGGAGGGGCTGGCTGGCTG<br>GAAGGCTTGGTGGCCTCTGACTTTTCTGTGGATCCAGGTCTCCCT<br>GAAAACCAGTGCCTTGGGGAGCTCGGTGGAAGAGGTAGAGCAG<br>TTGATTCGCAAGCACGAGGTCTTCCTGAAGGTTCTGACTGCCCA

GGACAAGAAGGTAATGGAGTCAGGAGGCCTGGGCGTGGTCAGA
GTGTGGTGGTTGGGAGAGAGTCCTGAAGGGGGTCACGGGGCGC
TTGGTGTGTTCCTGGAGGCAGTGCTGATGAGCCTGCAAGGCTGG
GGTCCTGGTTGGGGTTGACTGAGCAAGTTGTATGTGTGGCTCAA
GAAGAGGGCCCAGGGGGAGATGGGCCACACTGCAGAGACCCA
CACGGGGCATTTGGCTCTGTCCACACCGTGTGGTCCTTGGGGAG
CAGACTGGTCTGGCTTGTGGCTGGGAGGATCAGATACCCCTCAG
CCATGTGGGGCTGAGGAAAGGGCTCCGGGGCCCTCAAAGAAAG
GACTGTGAGCCCTGCAGGCCAGGCCCTGCCCCAGCCCAGGGTG
GGAGAGCAGCAGCTGTGCCCCTGCCCCACAGGAGGCAGCCCTG
CGTGAGCGGCTGAAGACGCTCCGGCGCCCCCGGGTGCGGGAC
CGGCTTCCCATCCTGCTGCAGCGCCGGAT

mir-4310 promoter mut A

GGAGGAGAGTTCGCAAGAGCCTAGCAGTGGCCCGCTGAAGCTC
AGTGCCCACCAGTGGCTCGCCCGCCTCGTGGAGGCCCGGGAGA
AGCTGTGGCAGCAGGCCACCCAGCTGGGGCAGCAGGCACTTCT
TGCTGCAGGGACACCCACCAAGGAGGTGGGCCCCCTCCCCTGG
TGCCCCCACATACCCTAGCTGACCAAGCCTGTGCCTGGCCACCT
GGTGCCCCGTCCTACTGTGCCATGCTGGCCTCTCCTCAGGAGCT
TCCCTCAGGATCCAGTCCTCACCTCACCCACACCACCCTCCCCA
GCTGAGGCTCCTCATGTGTCCATCCCCAGGTCCAGGAAGAGCTT
CGAGCCCTGCAGGACCAGCGGGACCAGGTGTATCAGACCTGGG
CACGGAAGCAAGAGAGGCTGCAGGCCGAGCAGCAGGAGCAGC
TCTTCCTCAGAGAGTGCGGCCGCCTGGAGGAGATCCTCGCGGC
CCAGGAGGCAGGTCCCCTCCCGCCCTCACACTCCGCTCAGCCTC
CTGCTCCTCCTTTCTATTCTCCTCTCTGCTCAGTCCCTTTCCTCCT
GCCTCTCTGGCTTTTTCTCTAATATGAGGCAGCTGTGCACAAGTC
CTTCCAGGTGGCCATGGTGAAGTGCTGAGGCCCCTGAGCGTCC
ACTGACATCCCCAGCCCCTATCCCAGACTGAGCCCCGAGGAAG
TTGGCCAGAGGCTGGTCATGCTCCCCACATGGTGCAGAGGTTGA
GGTATCGCAGCCTCCCAACCTGGACTCCCACTCCTCACAGCTCC
ATGCCAAGCTCAGTGGGCACTCCTAAGGCCCTGCCATACCCAGA
GTGCCCATGGGCACAGACTGCAGCTGGAGGGGCTGGCTGGCTG
GAAGGCTTGGTGGCCTCTGACTTTTCTGTGGATCCAGGTCTCCCT
GAAAACCAGTGCCTTGGGGAGCTCGGTGGAAGAGGTAGAGCAG
TTGATTCGCAAGCACGAGGTCTTCCTGAAGGTTCTGACTGCCCA
GGACAAGAAGGTAATGGAGTCAGGAGGCCTGGGCGTGGTCAGA
GTGTGGTGGTTGGGAGAGAGTCCTGAAGGGGGTCACGGGGCGC
TTGGTGTGTTCCTGGAGGCAGTGCTGATGAGCCTGCAAGGCTGG
GGTCCTGGTTGGGGTTGACTGAGCAAGTTGTATGTGTGGCTCAA
GAAGAGGGCCCAGGGGGAGATGGGCCACACTGCAGAGACCCA
CACGGGGCATTTGGCTCTGTCCACACCGTGTGGTCCTTGGGGAG
CAGACTGGTCTGGCTTGTGGCTGGGAGGATCAGATACCCCTCAG
CCATGTGGGGCTGAGGAAAGGGCTCCGGGGCCCTCAAAGAAAG
GACTGTGAGCCCTGCAGGCCAGGCCCTGCCCCAGCCCAGGGTG

	GGAGAGCAGCAGCTGTGCCCCTGCCCCACAGGAGGCAGCCCTG<br>CGTGAGCGGCTGAAGACGCTCCGGCGCCCCCGGGTGCGGGAC<br>CGGCTTCCCATCCTGCTGCAGCGCCGGAT
mir-4310 promoter<br>mut B	GGAGGAGAGTTCGCAAGAGCCTAGCAGTGGCCCGCTGAAGCTC<br>AGTGCCCACCAGTGGCTCCGGGCGGAGCTGGAGGCCCGGGAG<br>AAGCTGTGGCAGCAGGCCACCCAGCTGGGGCAGCAGGCACTTC<br>TTGCTGCAGGGACACCCACCAAGGAGGTGGGCCCCCTCCCCTG<br>GTGCCCCCACATACCCTAGCTGACCAAGCCTGTGCCTGGCCACC<br>TGGTGCCCCGTCCTACTGTGCCATGCTGGCCTCTCCTCAGGAGC<br>TTCCCTCAGGATCCAGTCCTCACCTCACCCACACCACCCTCCCC<br>AGCTGAGGCTCCTCATGTGTCCATCCCCAGGTCCAGGAAGAGCT<br>TCGAGCCCTGCAGGACCAGCGGGACCAGGTGTATCAGACCTGG<br>GCACGGAAGCAAGAGAGGCTGCAGGCCGAGCAGCAGGAGCAG<br>CTCTTCCTCAGAGAGTGCGGCCGCCTGGAGGAGATCCTCGCGG<br>CCCAGGAGGCAGGTCCCGAGGGCGGGACACACTCCGCTCAGC<br>CTCCTGCTCCTCCTTTCTATTCTCCTCTCTGCTCAGTCCCTTTCCTC<br>CTGCCTCTCTGGCTTTTTCTCTAATATGAGGCAGCTGTGCACAAG<br>TCCTTCCAGGTGGCCATGGTGAAGTGCTGAGGCCCCTGAGCGTC<br>CACTGACATCCCCAGCCCCTATCCCAGACTGAGCCCCGAGGAA<br>GTTGGCCAGAGGCTGGTCATGCTCCCCACATGGTGCAGAGGTTG<br>AGGTATCGCAGCCTCCCAACCTGGACTCCCACTCCTCACAGCTC<br>CATGCCAAGCTCAGTGGGCACTCCTAAGGCCCTGCCATACCCAG<br>AGTGCCCATGGGCACAGACTGCAGCTGGAGGGGCTGGCTGGCT<br>GGAAGGCTTGGTGGCCTCTGACTTTTCTGTGGATCCAGGTCTCC<br>CTGAAAACCAGTGCCTTGGGGAGCTCGGTGGAAGAGGTAGAGC<br>AGTTGATTCGCAAGCACGAGGTCTTCCTGAAGGTTCTGACTGCC<br>CAGGACAAGAAGGTAATGGAGTCAGGAGGCCTGGGCGTGGTCA<br>GAGTGTGGTGGTTGGGAGAGAGTCCTGAAGGGGGTCACGGGGC<br>GCTTGGTGTGTTCCTGGAGGCAGTGCTGATGAGCCTGCAAGGCT<br>GGGGTCCTGGTTGGGGTTGACTGAGCAAGTTGTATGTGTGGCTC<br>AAGAAGAGGGCCCAGGGGGAGATGGGCCACACTGCAGAGACC<br>CACACGGGGCATTTGGCTCTGTCCACACCGTGTGGTCCTTGGGG<br>AGCAGACTGGTCTGGCTTGTGGCTGGGAGGATCAGATACCCCTC<br>AGCCATGTGGGGCTGAGGAAAGGGCTCCGGGGCCCTCAAAGAA<br>AGGACTGTGAGCCCTGCAGGCCAGGCCCTGCCCCAGCCCAGGG<br>TGGGAGAGCAGCAGCTGTGCCCCTGCCCCACAGGAGGCAGCCC<br>TGCGTGAGCGGCTGAAGACGCTCCGGCGCCCCCGGGTGCGGGA<br>CCGGCTTCCCATCCTGCTGCAGCGCCGGAT
mir-4310 promoter<br>mut A+B	GGAGGAGAGTTCGCAAGAGCCTAGCAGTGGCCCGCTGAAGCTC<br>AGTGCCCACCAGTGGCTCGCCCGCCTCGTGGAGGCCCGGGAGA<br>AGCTGTGGCAGCAGGCCACCCAGCTGGGGCAGCAGGCACTTCT<br>TGCTGCAGGGACACCCACCAAGGAGGTGGGCCCCCTCCCCTGG<br>TGCCCCCACATACCCTAGCTGACCAAGCCTGTGCCTGGCCACCT<br>GGTGCCCCGTCCTACTGTGCCATGCTGGCCTCTCCTCAGGAGCT

TCCCTCAGGATCCAGTCCTCACCTCACCCACACCACCCTCCCCA
GCTGAGGCTCCTCATGTGTCCATCCCCAGGTCCAGGAAGAGCTT
CGAGCCCTGCAGGACCAGCGGGACCAGGTGTATCAGACCTGGG
CACGGAAGCAAGAGAGGCTGCAGGCCGAGCAGCAGGAGCAGC
TCTTCCTCAGAGAGTGCGGCCGCCTGGAGGAGATCCTCGCGGC
CCAGGAGGCAGGTCCCGAGGGCGGGACACACTCCGCTCAGCCT
CCTGCTCCTCCTTTCTATTCTCCTCTCTGCTCAGTCCCTTTCCTCCT
GCCTCTCTGGCTTTTTCTCTAATATGAGGCAGCTGTGCACAAGTC
CTTCCAGGTGGCCATGGTGAAGTGCTGAGGCCCCTGAGCGTCC
ACTGACATCCCCAGCCCCTATCCCAGACTGAGCCCCGAGGAAG
TTGGCCAGAGGCTGGTCATGCTCCCCACATGGTGCAGAGGTTGA
GGTATCGCAGCCTCCCAACCTGGACTCCCACTCCTCACAGCTCC
ATGCCAAGCTCAGTGGGCACTCCTAAGGCCCTGCCATACCCAGA
GTGCCCATGGGCACAGACTGCAGCTGGAGGGGCTGGCTGGCTG
GAAGGCTTGGTGGCCTCTGACTTTTCTGTGGATCCAGGTCTCCCT
GAAAACCAGTGCCTTGGGGAGCTCGGTGGAAGAGGTAGAGCAG
TTGATTCGCAAGCACGAGGTCTTCCTGAAGGTTCTGACTGCCCA
GGACAAGAAGGTAATGGAGTCAGGAGGCCTGGGCGTGGTCAGA
GTGTGGTGGTTGGGAGAGAGTCCTGAAGGGGGTCACGGGGCGC
TTGGTGTGTTCCTGGAGGCAGTGCTGATGAGCCTGCAAGGCTGG
GGTCCTGGTTGGGGTTGACTGAGCAAGTTGTATGTGTGGCTCAA
GAAGAGGGCCCAGGGGGAGATGGGCCACACTGCAGAGACCCA
CACGGGGCATTTGGCTCTGTCCACACCGTGTGGTCCTTGGGGAG
CAGACTGGTCTGGCTTGTGGCTGGGAGGATCAGATACCCCTCAG
CCATGTGGGGCTGAGGAAAGGGCTCCGGGGCCCTCAAAGAAAG
GACTGTGAGCCCTGCAGGCCAGGCCCTGCCCCAGCCCAGGGTG
GGAGAGCAGCAGCTGTGCCCCTGCCCCACAGGAGGCAGCCCTG
CGTGAGCGGCTGAAGACGCTCCGGCGCCCCCGGGTGCGGGAC
CGGCTTCCCATCCTGCTGCAGCGCCGGAT

Supplementary Table 2. A list of antibodies used for Western blot, IHC staining, CHIP, EMSA

Name of antibody	Cat. No	Company	Species	Dilution
ZEB1	21544-1-AP	PTG	Rabbit	1:1000(WB)
N-cadherin	22018-1-AP	PTG	Rabbit	1:1000(WB)
E- cadherin	60335-1-Ig	PTG	Mouse	1:1000(WB)
PI3K	60225-1-Ig	PTG	Mouse	1:1000(WB)
p-PI3K	4228	CST	Rabbit	1:1000(WB)
AKT	4691	CST	Rabbit	1:1000(WB)
p-AKT	4060	CST	Rabbit	1:1000(WB)
p21	2947	CST	Rabbit	1:1000(WB)
P27	3686	CST	Rabbit	1:1000(WB)
Ki67	Ab16667	Abcam	Rabbit	1:100 (IHC)
PCNA	13110	CST	Rabbit	1:1000 (IHC)

PTEN	60300-1-Ig	PTG	Mouse	1:1000 (WB) 1:500(IHC)
sp1	9389	CST	Rabbit	1:1000 (WB, IHC)
GAPDH	60004-1-Ig	PTG	Mouse	1:5000 (WB)
β-actin	60008-1-Ig	PTG	Mouse	1:5000 (WB)
