# Supplementary figures and images for: MiR-4310 induced by SP1 targets PTEN to promote glioma progression
Source: Cancer Cell Int. 2020 Dec 17;20:567. doi: 10.1186/s12935-020-01650-9 (PMC7745362; doi:10.1186/s12935-020-01650-9)

A

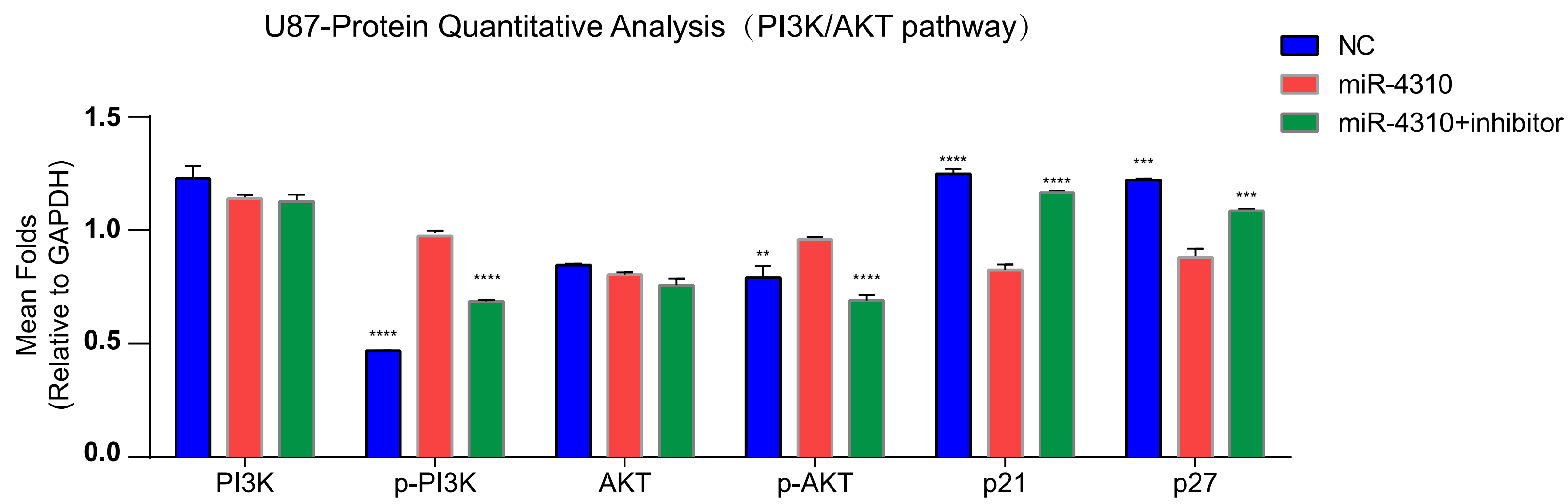

B

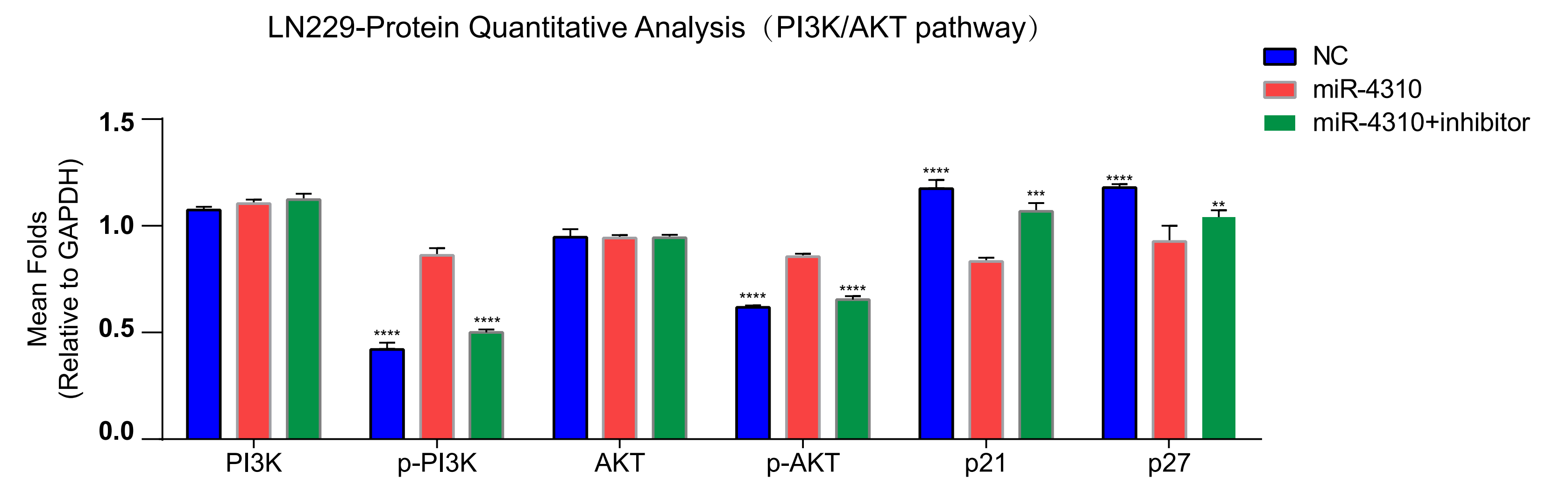

C

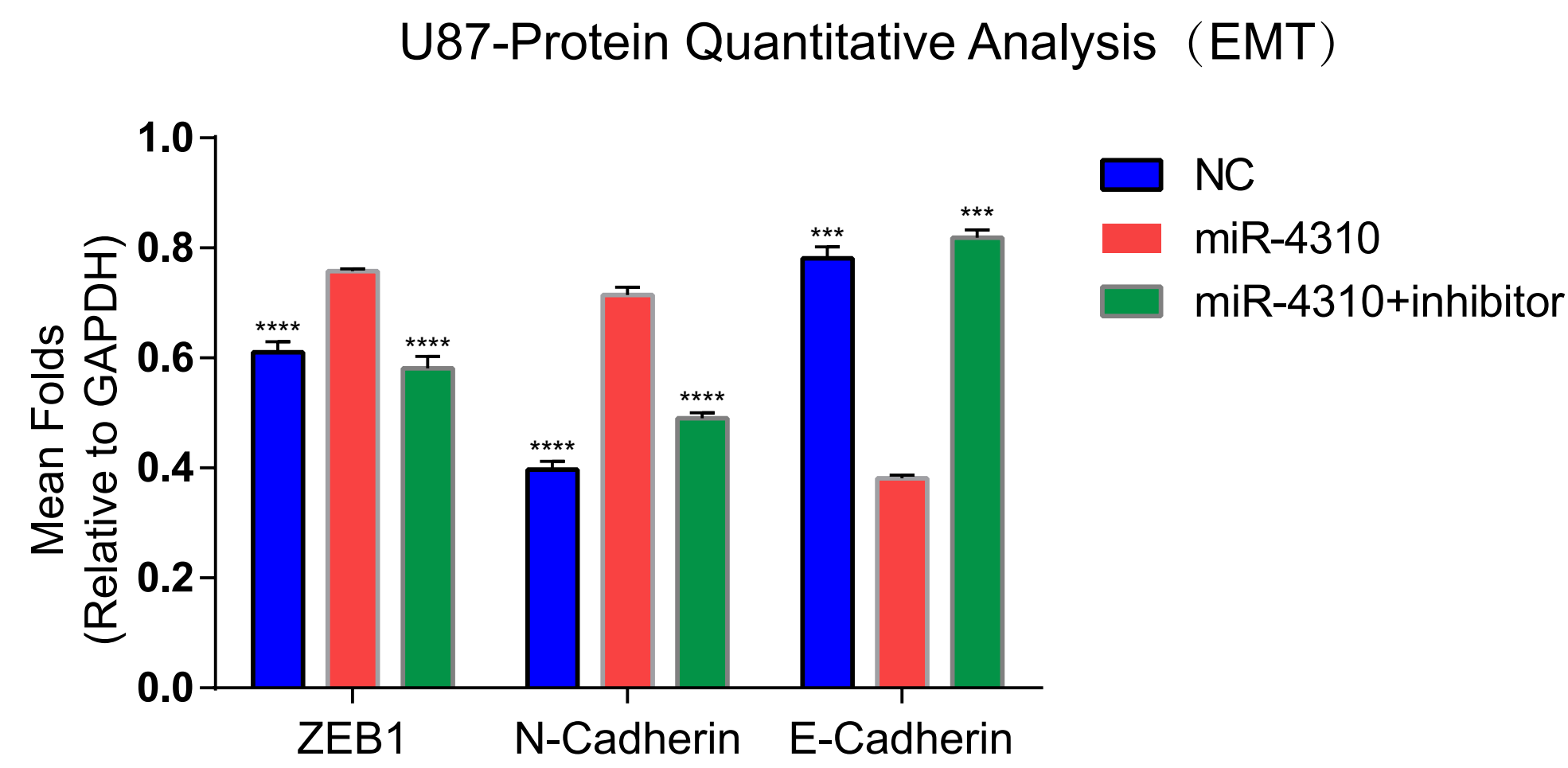

D

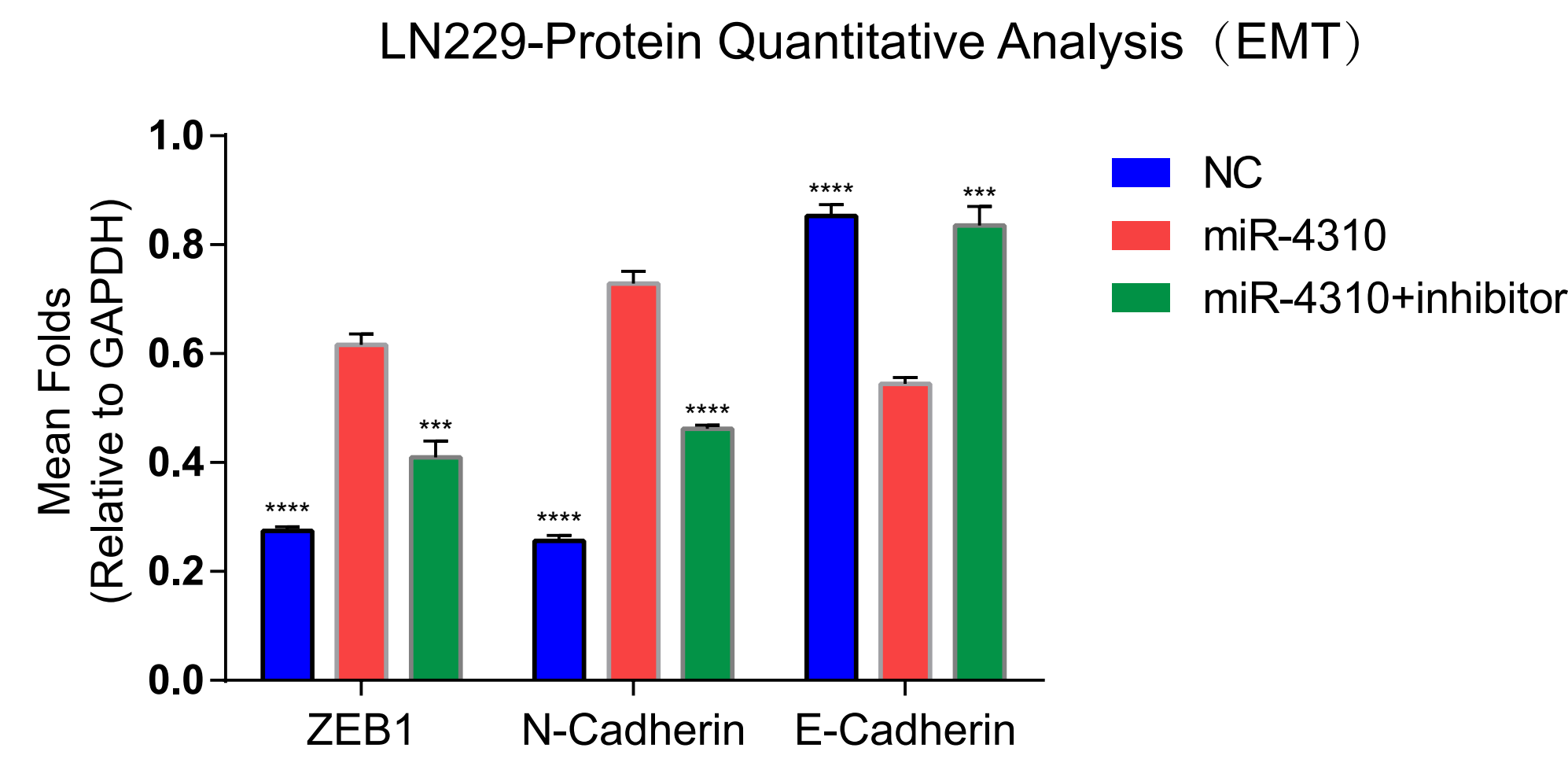

E

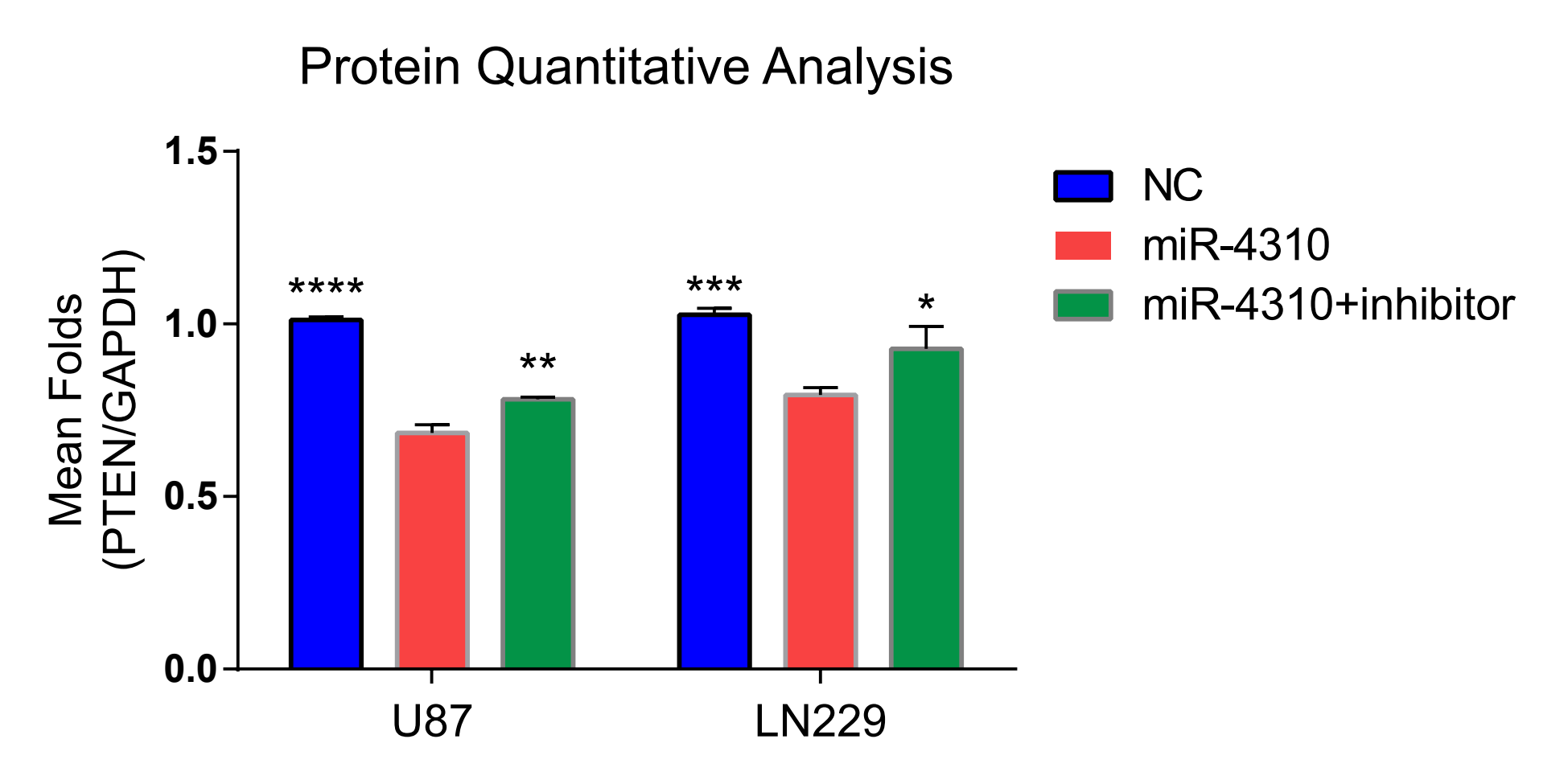

F

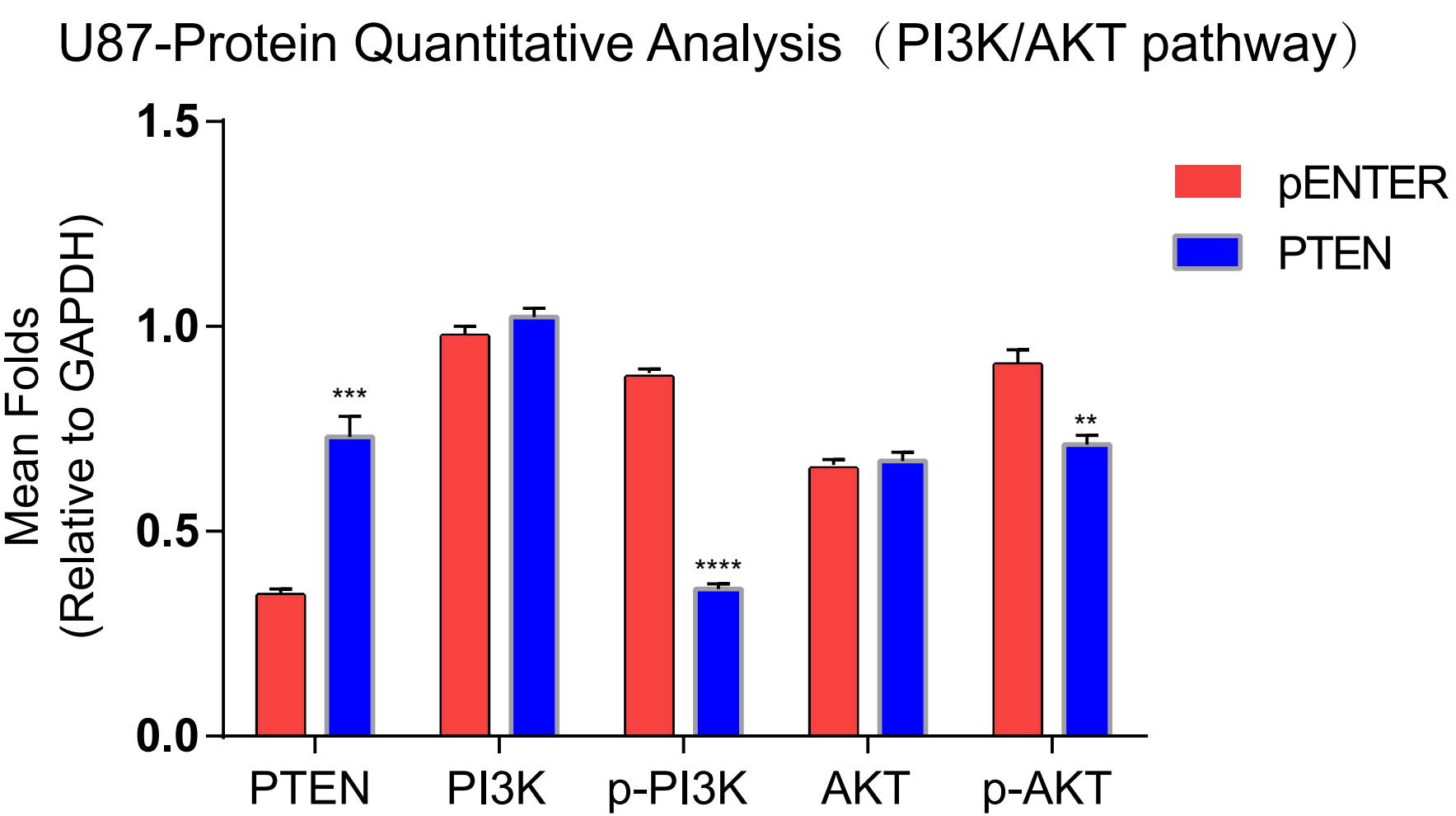

G

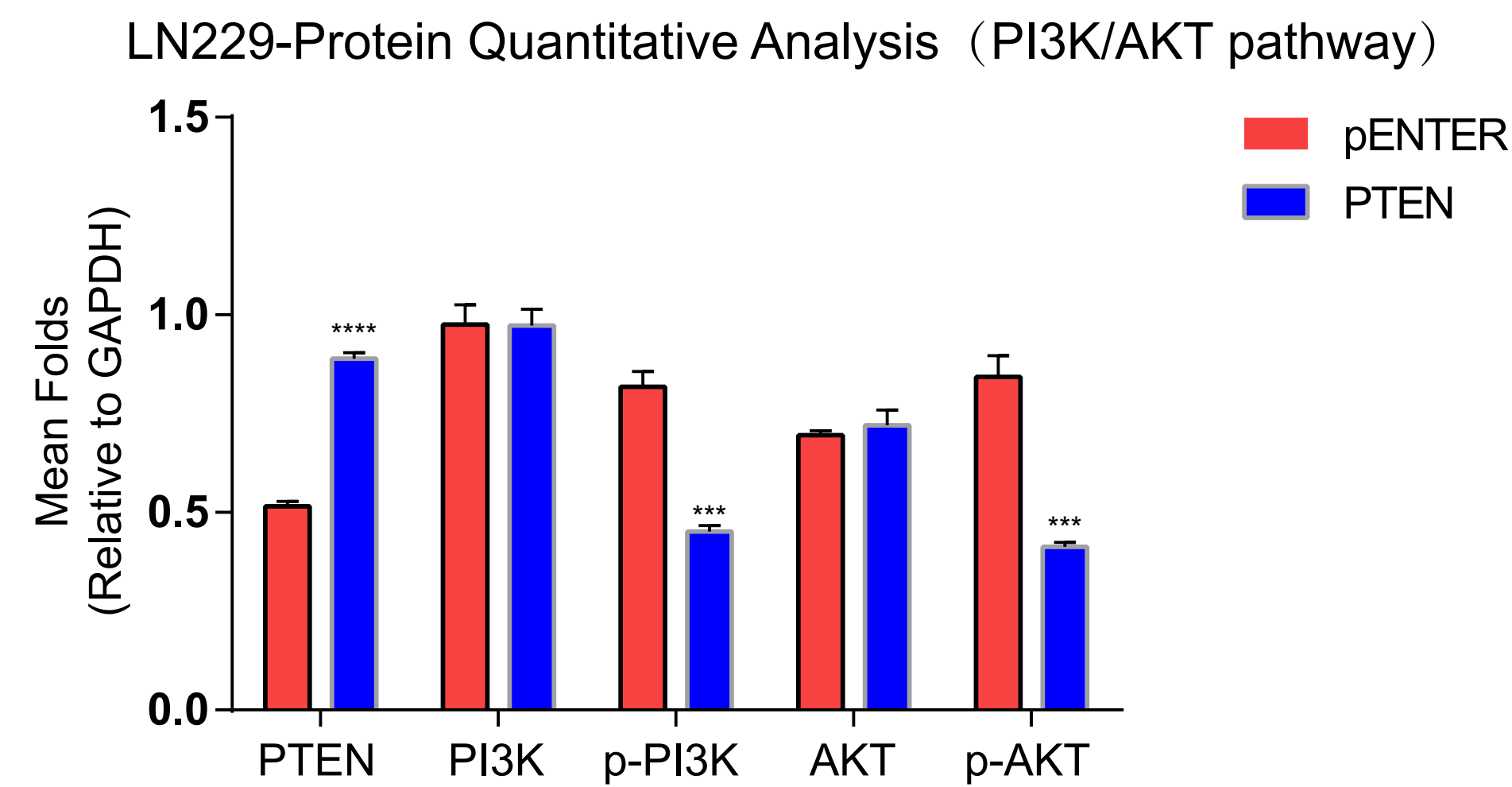

H

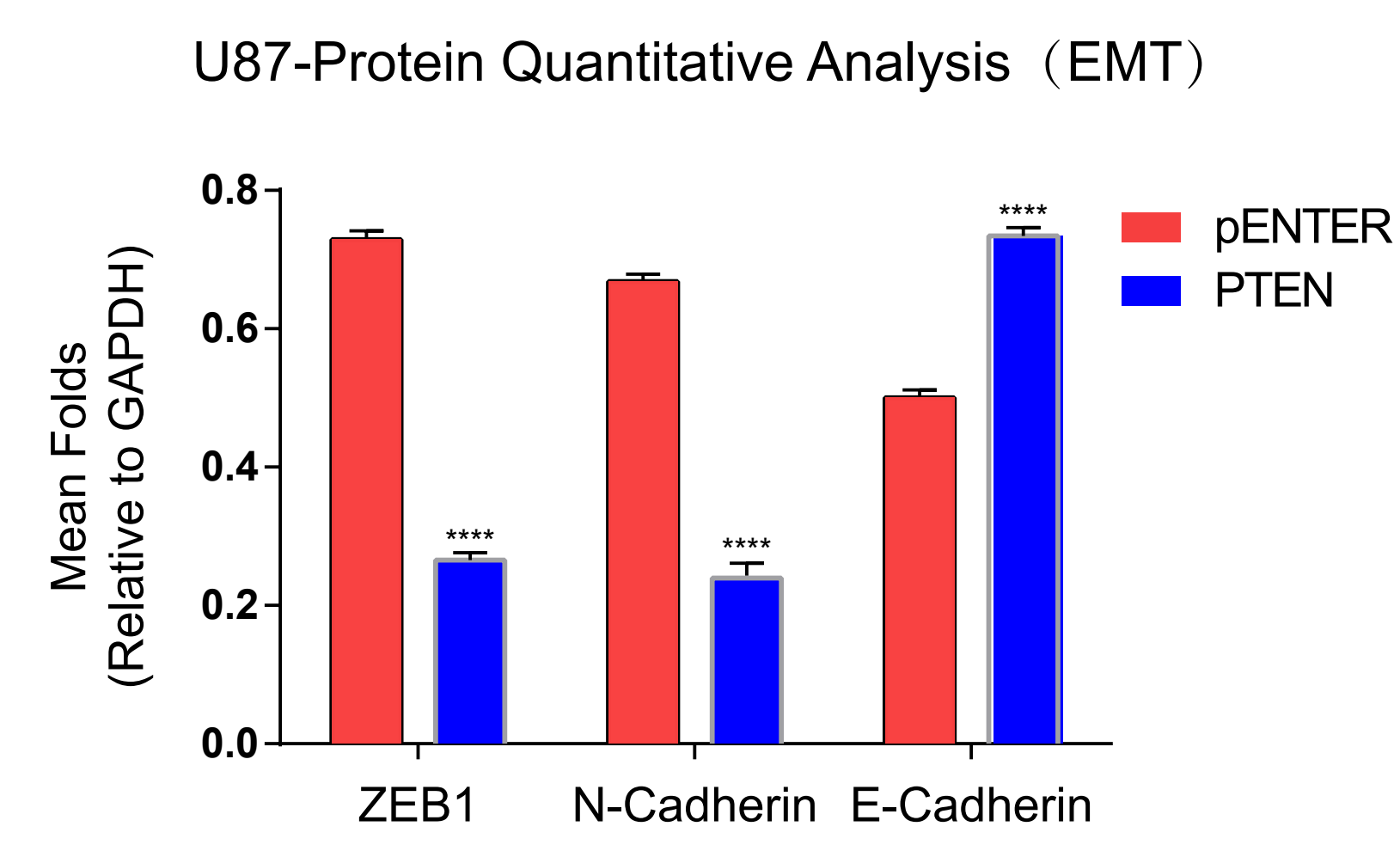

I

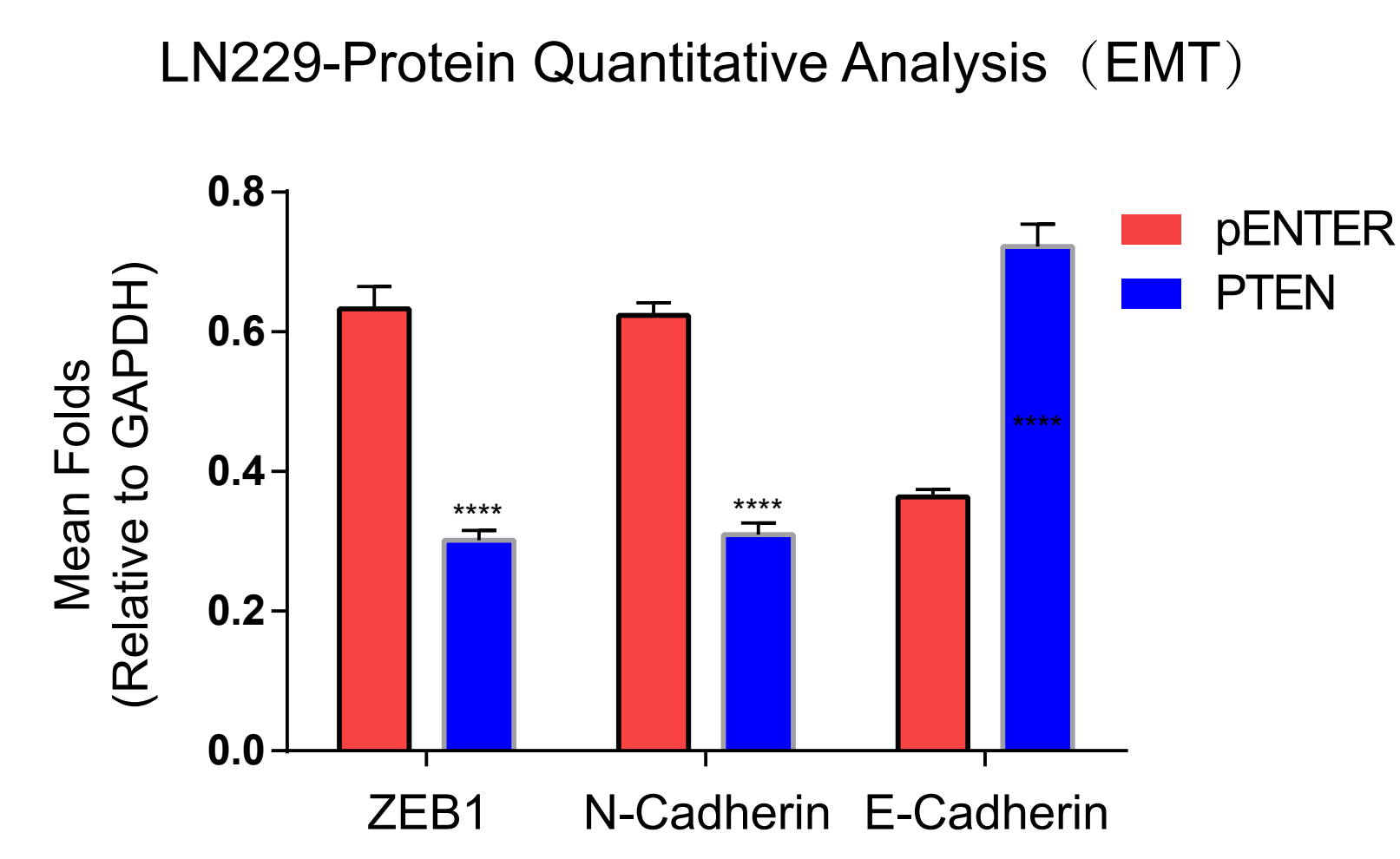

J

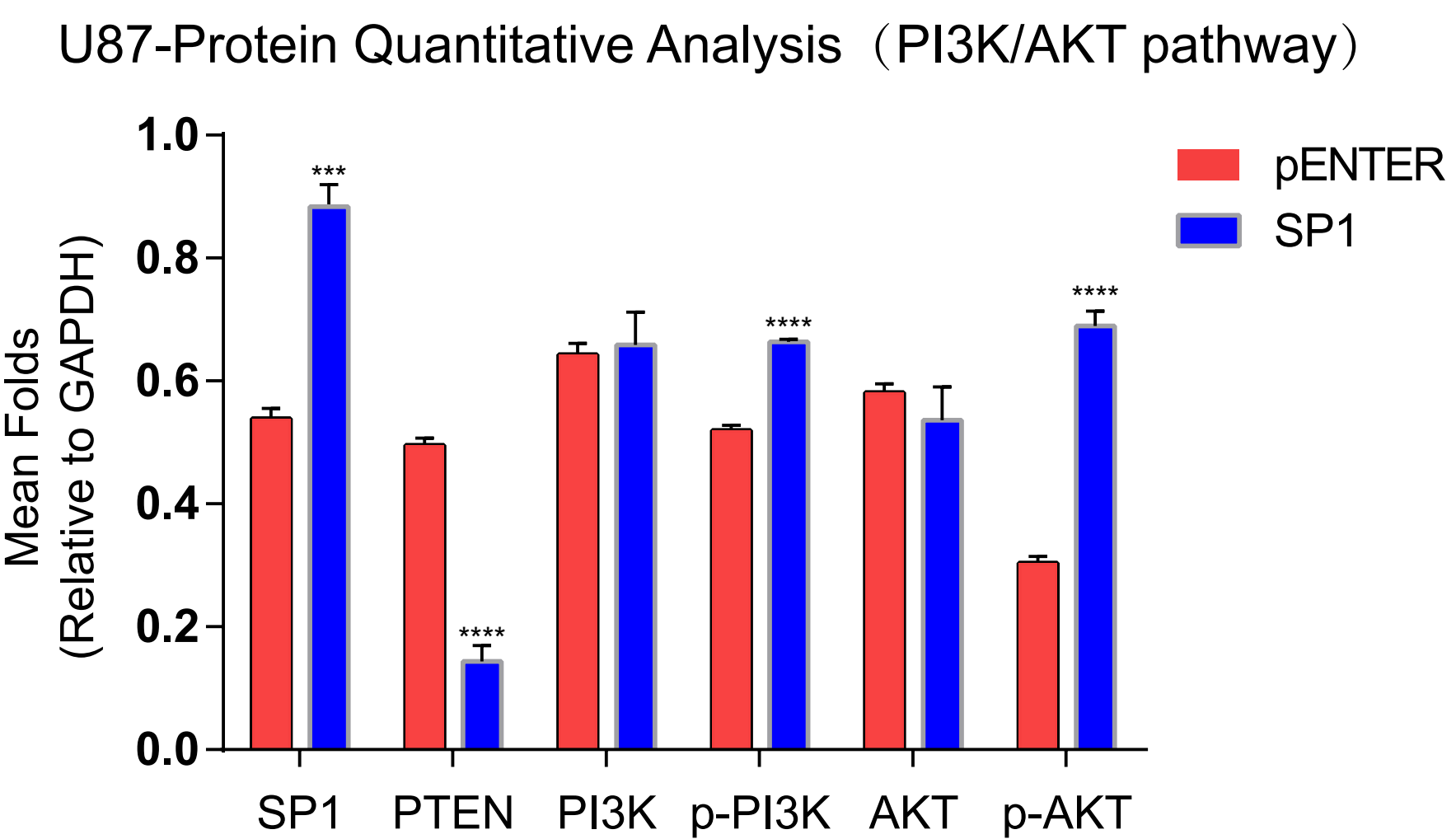

K

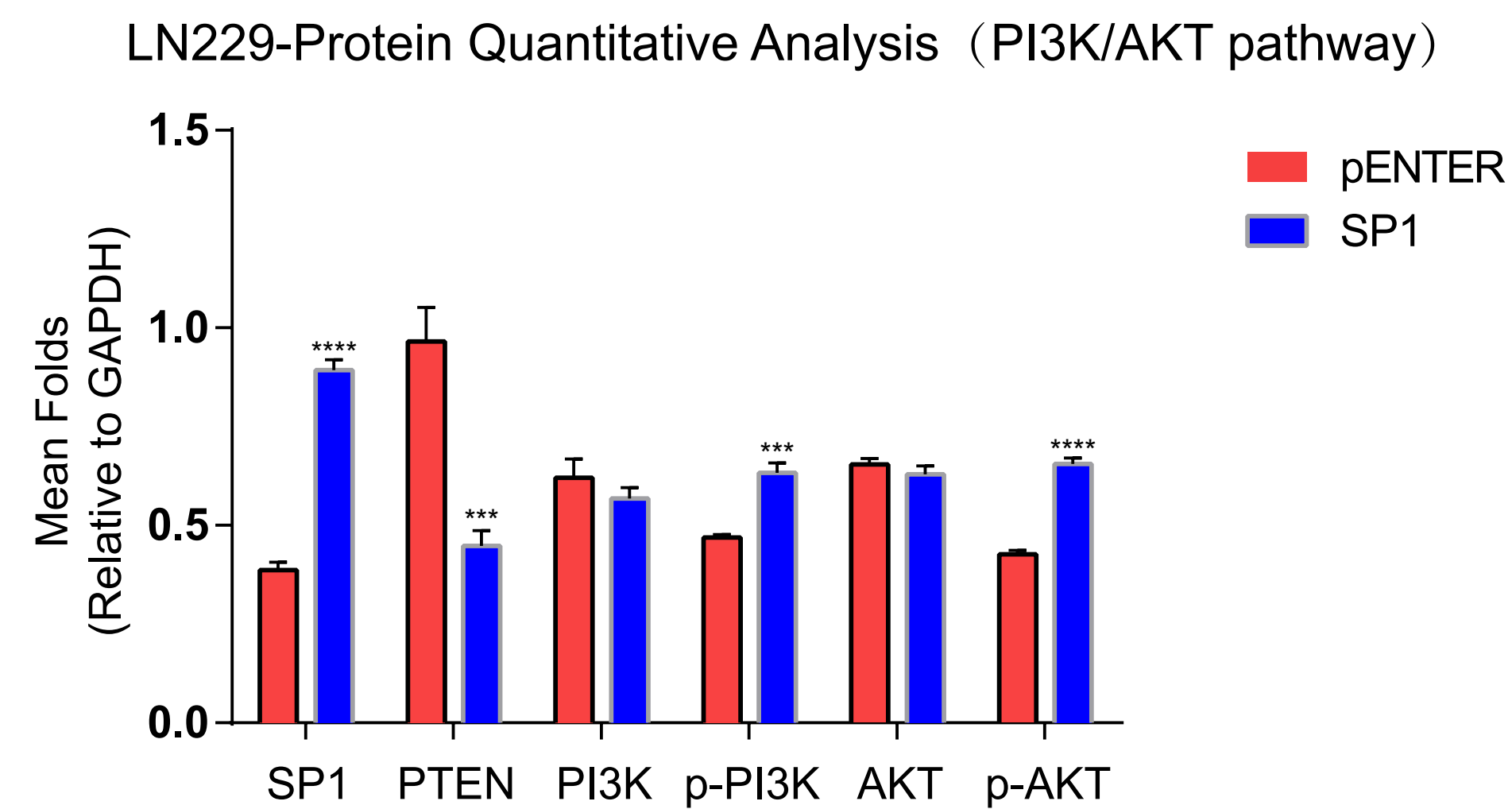

L

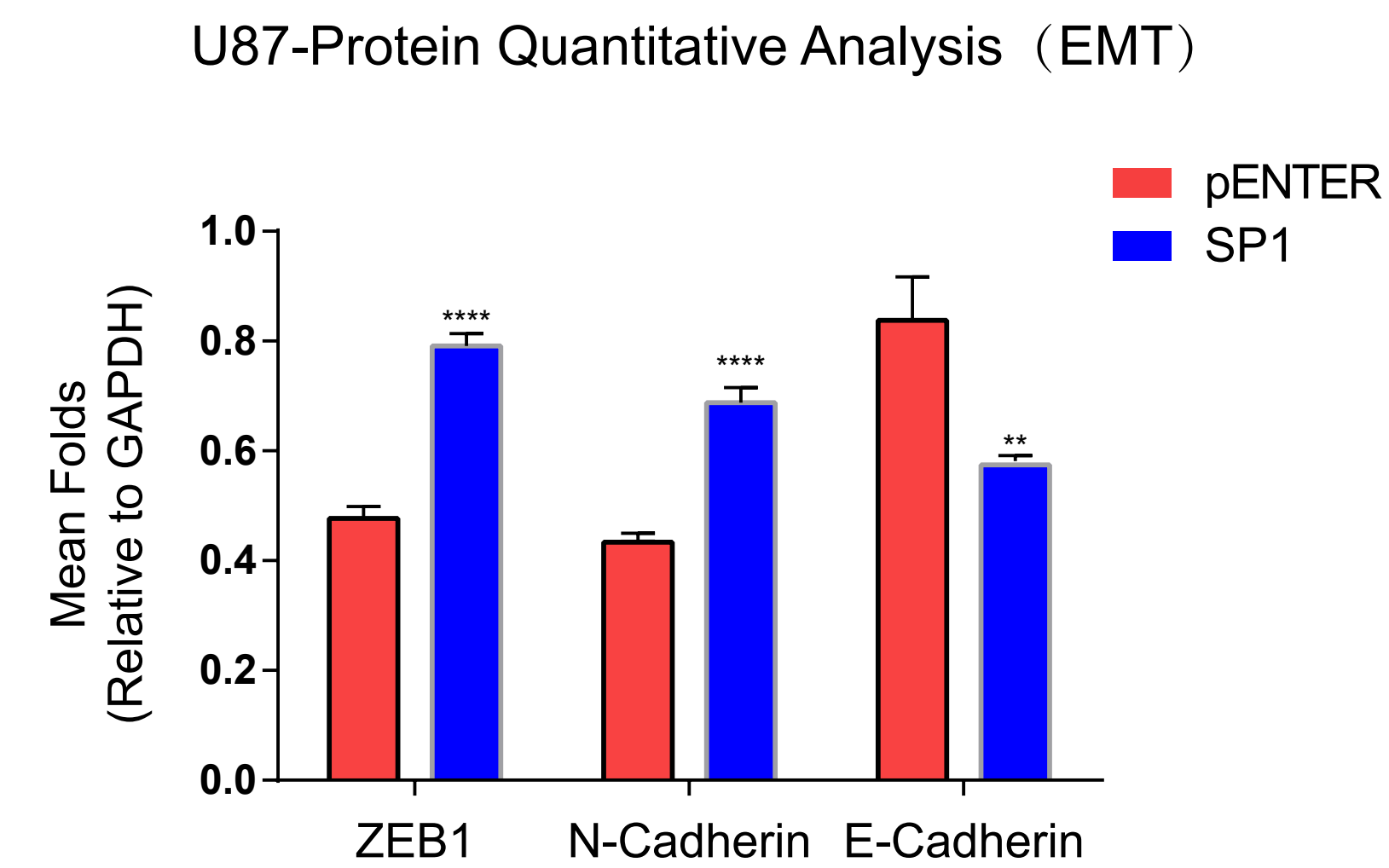

M

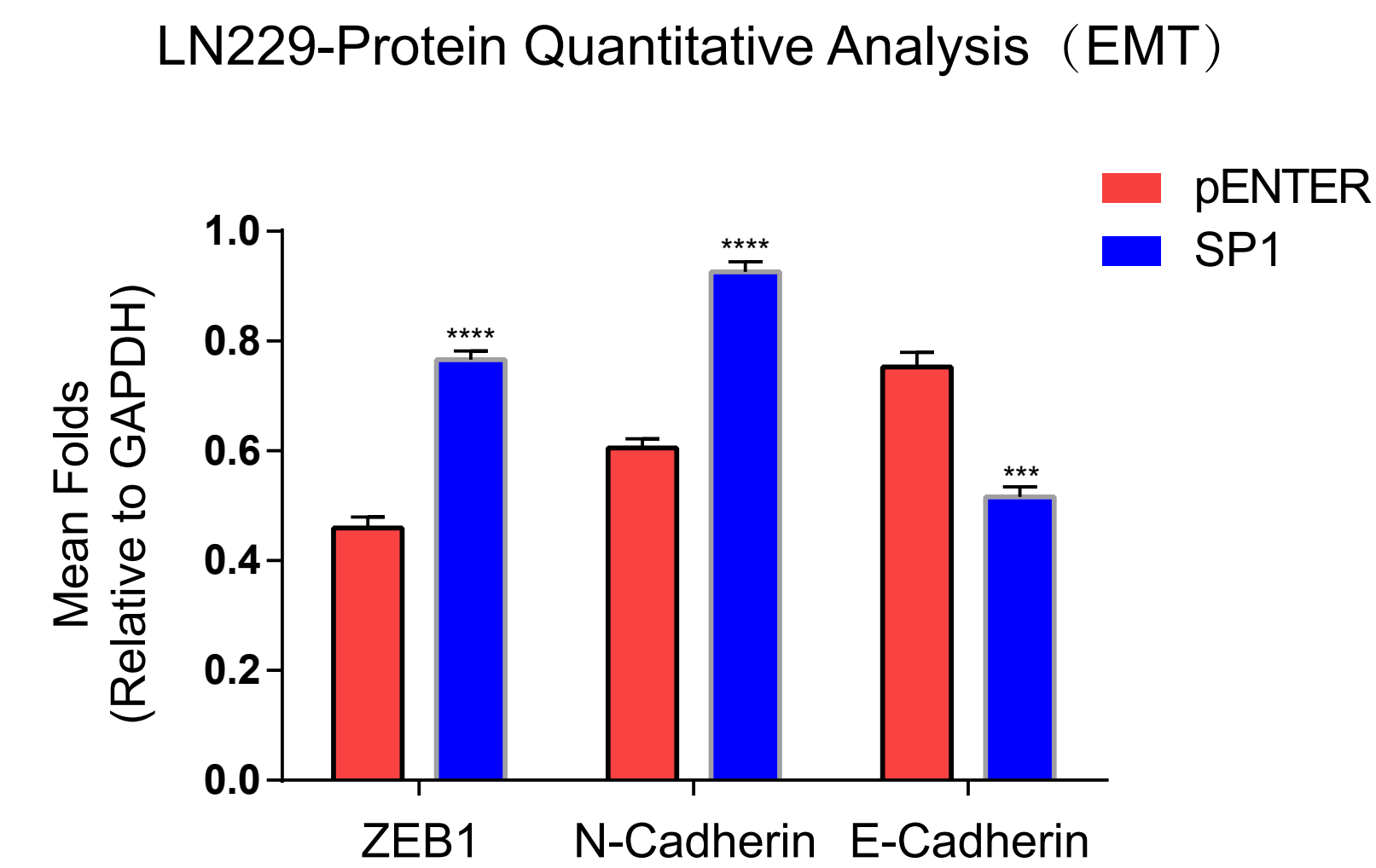

Supplement: Supplementary file 2 — Additional file 2: Figure S1. a–d Protein quantitative analysis for Fig. 1g, h. e Protein quantitative analysis for Fig. 3c. f–i Protein quantitative analysis for Fig. 4e, f. j–m Protein quantitative analysis for Fig. 5c, d. [file 12935_2020_1650_MOESM2_ESM.pdf]

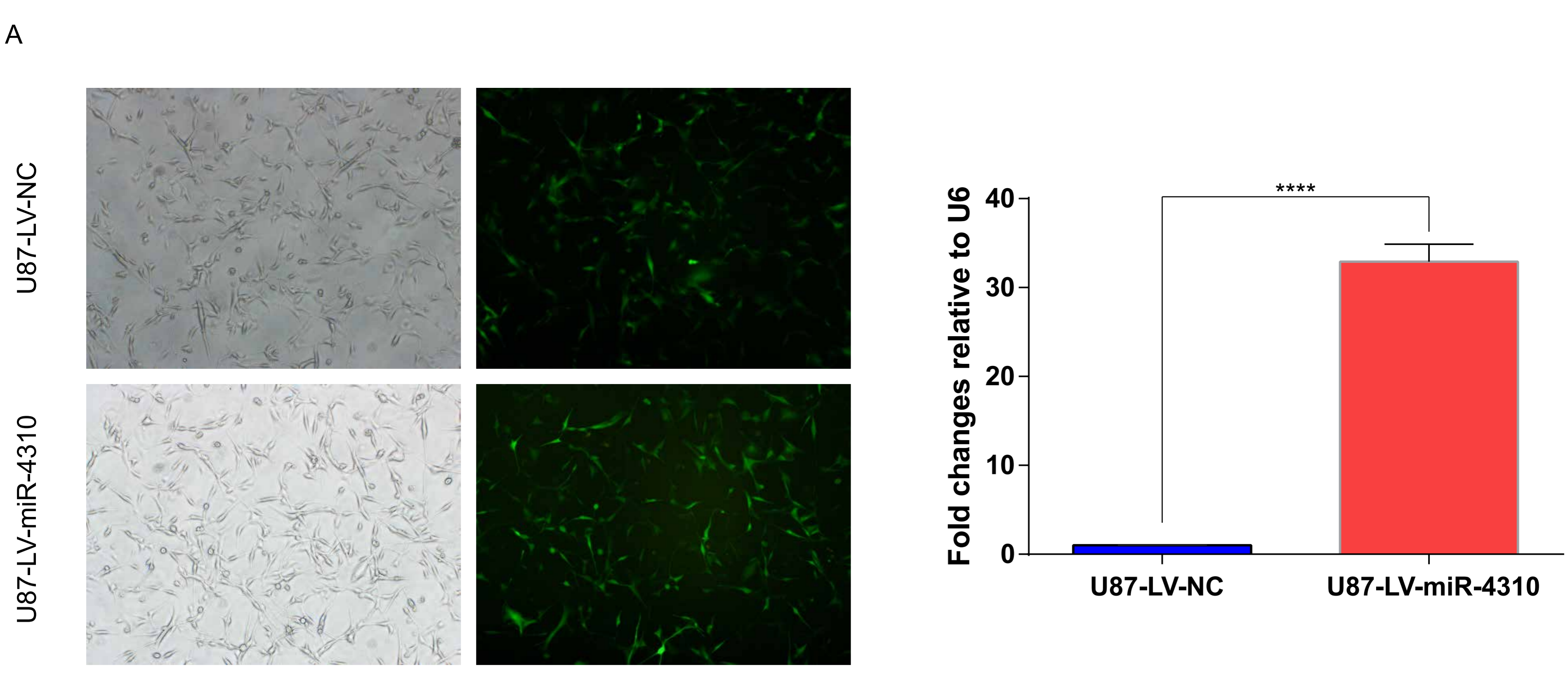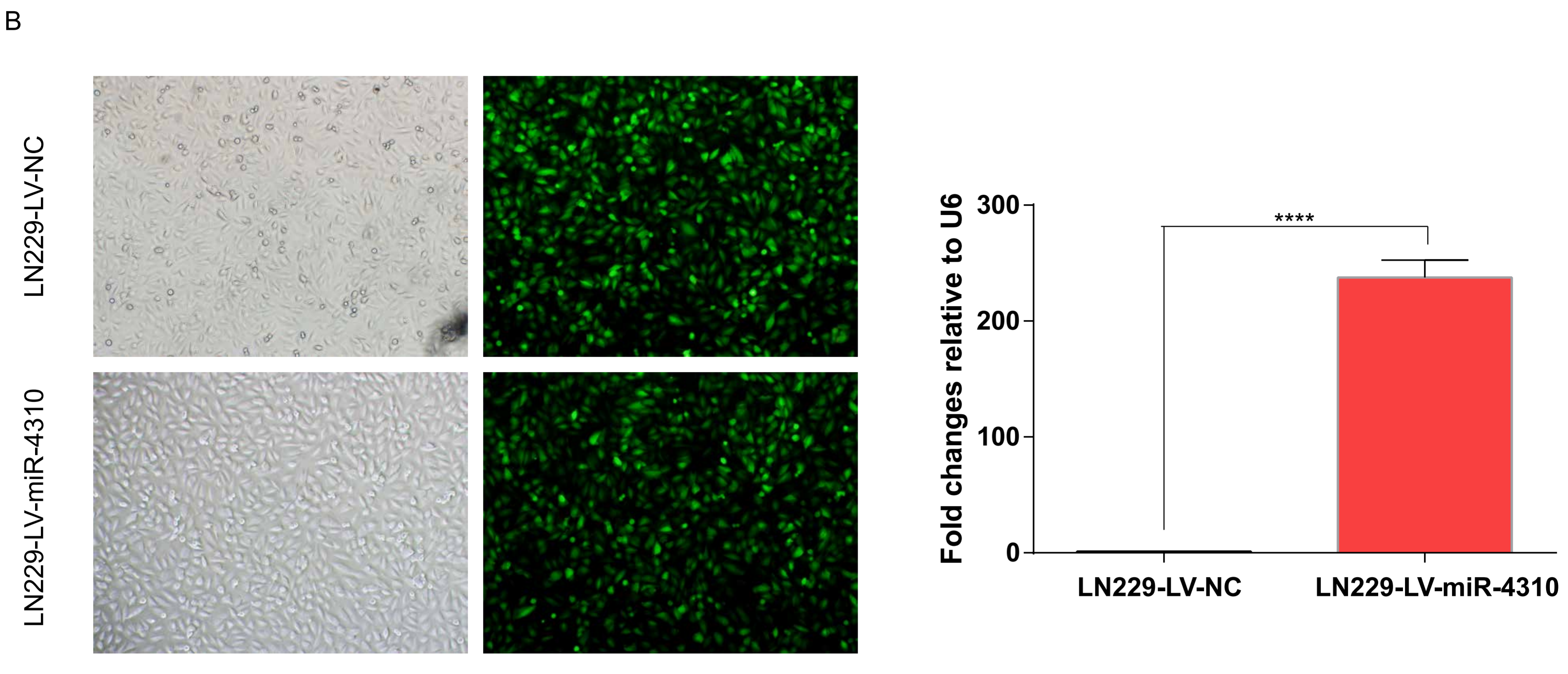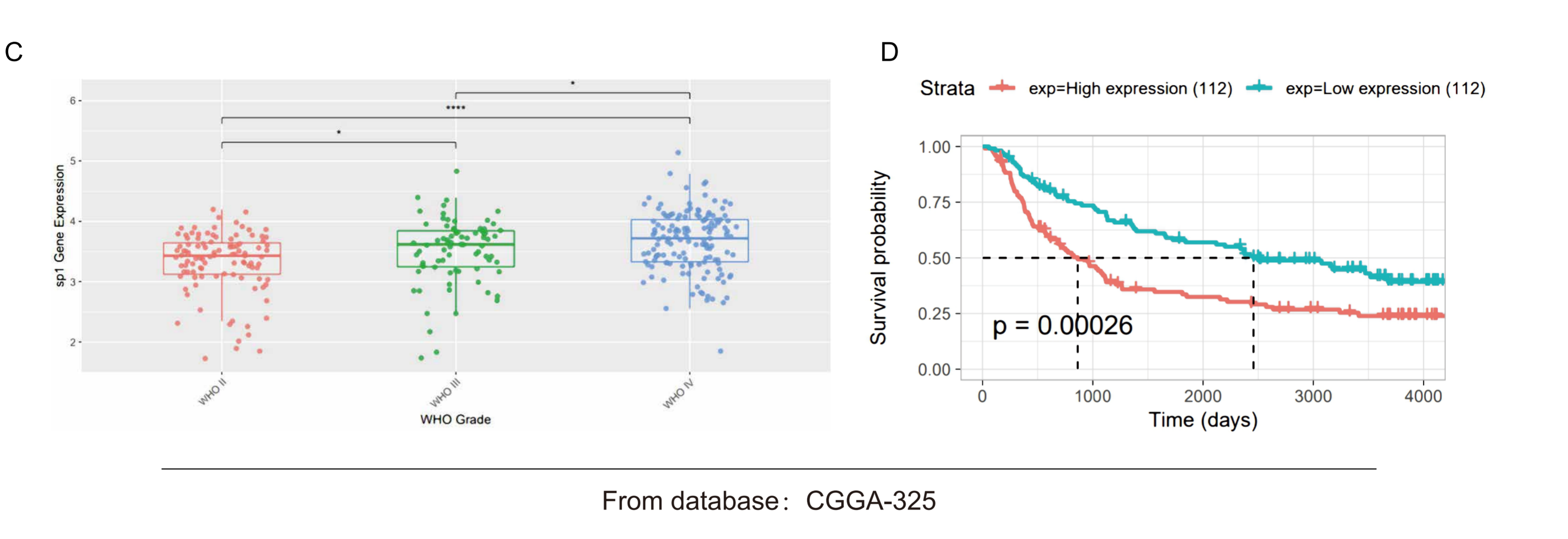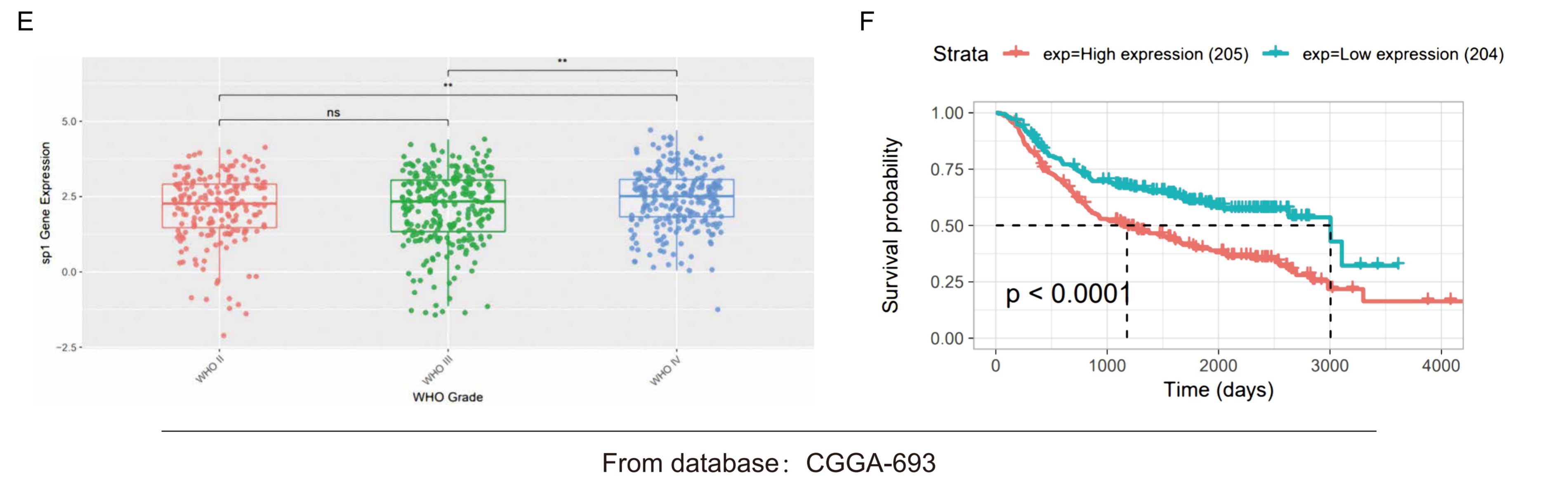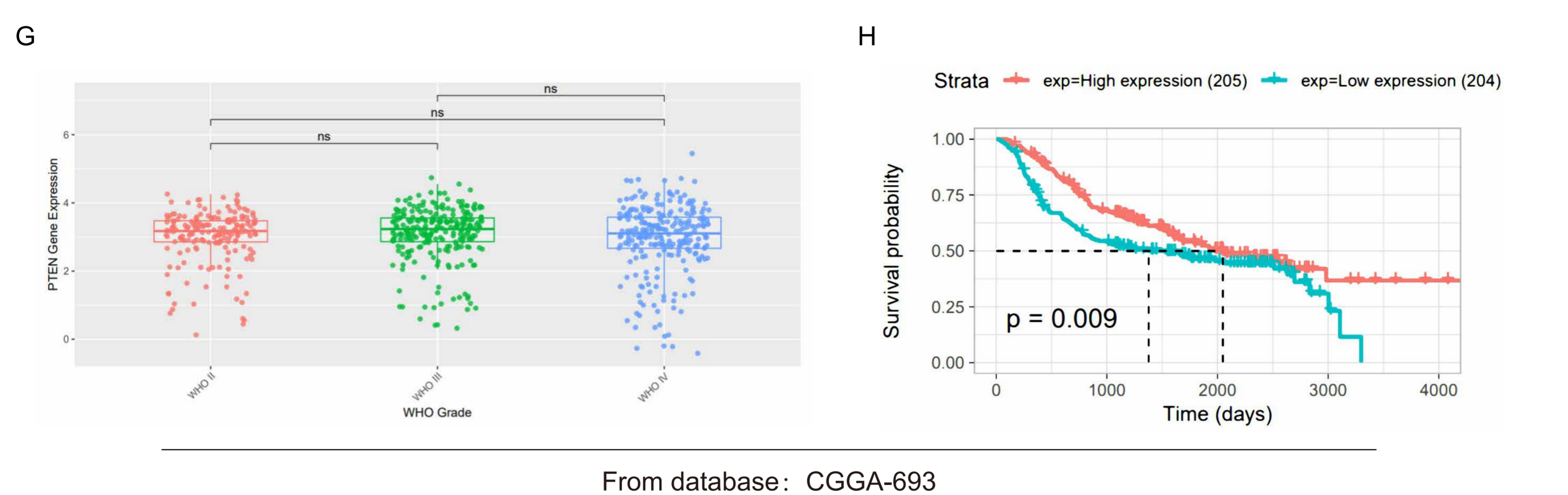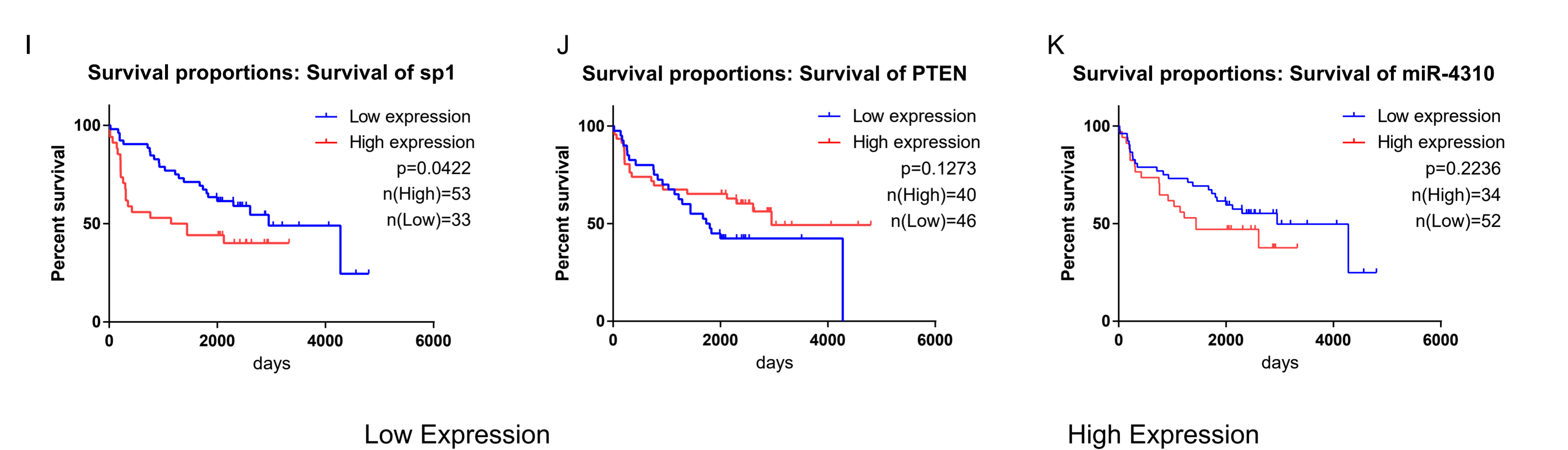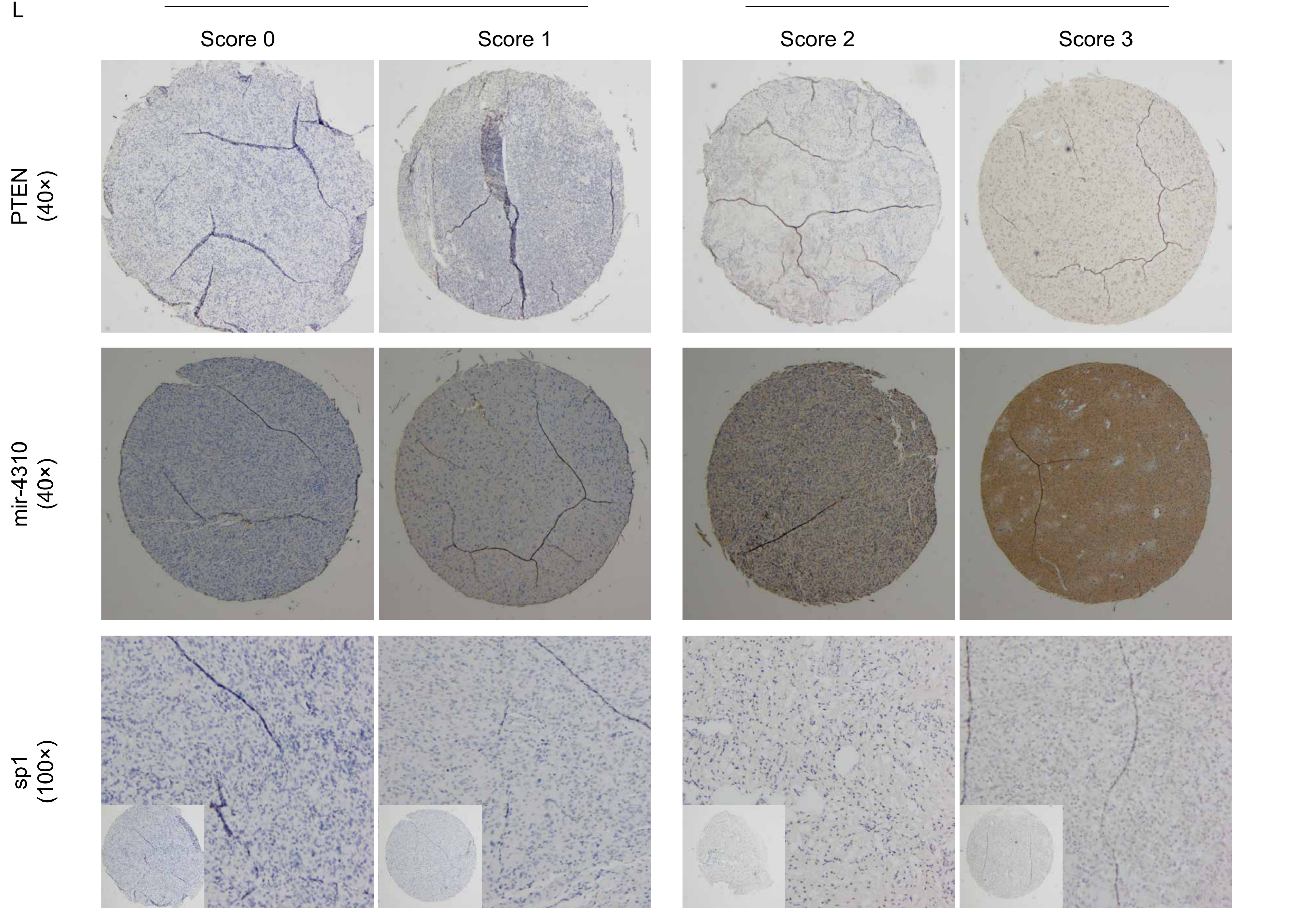

Supplement: Supplementary file 3 — Additional file 3: Figure S2. a, b Fluorescence analysis and qPCR assay to confirm the efficiency of U87 and LN229 after transfection with lentivirus. c-h Expression of SP1 and PTEN in each grade of glioma was shown, and Kaplan-Meier survival analysis based on SP1 and PTEN expression were performed in CGGA datasets. i-k Kaplan–Meier survival analysis of overall survival of 86 glioma patients on the basis of SP1, PTEN and miR-4310 expression levels were performed in Nanfang Hospital cohort. l The example diagram of high expression and low expression of miR-4310, PTEN and SP1 in ISH or IHC. [file 12935_2020_1650_MOESM3_ESM.pdf]
